# Adolescents show differential dysfunctions related to Alcohol and Cannabis Use Disorder severity in emotion and executive attention neuro-circuitries

**DOI:** 10.1016/j.nicl.2018.06.005

**Published:** 2018-06-05

**Authors:** Joseph Aloi, Karina S. Blair, Kathleen I. Crum, Harma Meffert, Stuart F. White, Patrick M. Tyler, Laura C. Thornton, Alita M. Mobley, Abraham D. Killanin, Kathryn O. Adams, Francesca Filbey, Kayla Pope, R. James R. Blair

**Affiliations:** a Center for Neurobehavioral Research, Boys Town National Research Hospital, Boys Town, NE, United States; b Department of Pharmacology and Experimental Neuroscience, University of Nebraska Medical Center, Omaha, NE, United States; c Department of Psychiatry, University of Nebraska Medical Center, Omaha, NE, United States; d Department of Psychiatry, Creighton University, Omaha, NE, United States; e Center for BrainHealth, School of Behavioral and Brain Sciences, University of Texas at Dallas, Dallas, TX, United States

**Keywords:** Adolescent, Alcohol Use Disorder, Amygdala, Cannabis Use Disorder, fMRI, Prefrontal cortex

## Abstract

Alcohol and cannabis are two substances that are commonly abused by adolescents in the United States and which, when abused, are associated with negative medical and psychiatric outcomes across the lifespan. These negative psychiatric outcomes may reflect the detrimental impact of substance abuse on neural systems mediating emotion processing and executive attention. However, work indicative of this has mostly been conducted either in animal models or adults with Alcohol and/or Cannabis Use Disorder (AUD/CUD). Little work has been conducted in adolescent patients. In this study, we used the Affective Stroop task to examine the relationship in 82 adolescents between AUD and/or CUD symptom severity and the functional integrity of neural systems mediating emotional processing and executive attention. We found that AUD symptom severity was *positively* related to amygdala responsiveness to emotional stimuli and *negatively* related to responsiveness within regions implicated in executive attention and response control (i.e., dorsolateral prefrontal cortex, anterior cingulate cortex, precuneus) as a function of task performance. In contrast, CUD symptom severity was unrelated to amygdala responsiveness but *positively* related to responsiveness within regions including precuneus, posterior cingulate cortex, and inferior parietal lobule as a function of task performance. These data suggest differential impacts of alcohol and cannabis abuse on the adolescent brain.

## Highlights

• Alcohol and cannabis are the most widely abused substances by adolescents.
• AUDIT scores are related to amygdala hyperactivity to emotional stimuli.
• AUDIT scores are related to hypoactivity in executive attention neuro-circuitry.
• CUDIT scores are related to hyperactivity in executive attention neuro-circuitry.

## 1 Introduction

Two of the most commonly abused substances by adolescents in the US are alcohol and cannabis (Miech et al., 2016). Notably, epidemiological evidence suggests that adolescent alcohol users are twice as likely to develop Alcohol Use Disorder (AUD) while adolescent cannabis users are over three times as likely to develop Cannabis Use Disorder (CUD) by age 26 than non-users (Winters and Lee, 2008). Furthermore, adolescents who initiate substance use face a more severe disease course and a greater likelihood of relapse (Babor et al., 1992). This may reflect the deleterious neurodevelopmental impact of substance abuse on the adolescent brain (Filbey et al., 2015; Squeglia et al., 2015), which is undergoing critical changes at this time (Goddings et al., 2014).

One neuro-circuitry undergoing development during adolescence that may be disrupted by substance abuse is the neuro-circuitry mediating emotional processing (Koob and Volkow, 2016). Animal work suggests that substance dependence leads to decreased striatal response to reward and increased amygdala responsiveness to stress (Koob and Volkow, 2016). In line with this, there have been reports of increased amygdala responses to negative images in alcohol dependent adults relative to controls (Gilman and Hommer, 2008), and in undergraduate students who also demonstrated relatively low ventral striatal responsiveness to reward (Nikolova et al., 2016). Additionally, there has been at least one report of increased amygdala responsiveness to angry relative to neutral faces in adolescents with mild cannabis use histories (group average: <5 times lifetime usage) (Spechler et al., 2015). However, other work has reported reduced amygdala responses to emotional relative to neutral faces in alcohol dependent adults (O'Daly et al., 2012) and in adult heavy cannabis smokers relative to healthy control adults (Gruber et al., 2010). In short, the human fMRI literature is somewhat inconsistent and focused on studies with adult participants.

A second putative neuro-circuitry disrupted by substance abuse is that mediating behavioral inhibition (Feldstein Ewing et al., 2014; Silveri et al., 2016; Spear, 2016); i.e., anterior cingulate/dorsomedial prefrontal cortices (ACC/dmPFC) and anterior insular cortex/inferior frontal gyrus (aIC/iFG; Criaud and Boulinguez, 2013). Moreover, substance abuse may also disrupt regions showing dense projections with ACC/dmPFC (i.e. dorsolateral prefrontal (dlPFC) and parietal cortices) which are critical for executive attention (Desimone and Duncan, 1995; Squire et al., 2013). Neuroimaging work has revealed that, relative to controls, undergraduate students and adults with heavy alcohol use histories show reduced ACC responses during NoGo trials relative to baseline (Ahmadi et al., 2013; Claus et al., 2013) and reduced dlPFC responses during successful, relative to unsuccessful, Stop trials during a Stop Signal Task (Li et al., 2009). Furthermore, ACC functional connectivity has been identified as a predictor of relapse in adults aged 18–50 with AUD (Zakiniaeiz et al., 2017). The literature in adolescents aged 18 and younger has been more mixed. One study reported an inverse relationship between prior alcohol consumption and aIC responses to incongruent relative to congruent trials during a Stroop task (Thayer et al., 2015). Another study which tracked youths from early to late adolescence reported that adolescents (ages 11–17) who later transitioned into heavy drinking showed *decreased* activity within middle frontal and parietal cortices in NoGo relative to Go trials prior to the onset of heavy drinking compared to controls who did not transition into heavy drinking (Norman et al., 2011; Wetherill et al., 2014). At a three-year follow-up after the onset of heavy drinking (at ages 14–21), adolescents who did transition to heavy drinking showed *increased* BOLD responses in these contrasts and brain regions relative to their baseline scans. However, participants who did not transition to heavy drinking showed *decreased* BOLD responses in these contrasts and brain regions relative to their baseline scans (Wetherill et al., 2014).

The empirical literature suggests a rather different relationship between cannabis usage and brain regions implicated in behavioral inhibition or executive attention, specifically *increased* (potentially compensatory) recruitment of these regions. In a Stroop task, adults with histories of heavy cannabis use showed increased ACC and dlPFC activity during interference trials relative to controls (Gruber and Yurgelun-Todd, 2005). Additionally, in a Multi-Source Interference Task, adults with histories of chronic cannabis smoking showed increased ACC recruitment during interference trials relative to control trials compared to healthy control subjects (Gruber et al., 2013). Furthermore, Filbey and Yezhuvath (2013) showed that cannabis-dependent adults showed greater connectivity between right frontal cortex and the substantia nigra/subthalamic nucleus network during successful inhibition on a Stop Signal task compared to non-dependent cannabis using adults. In a sample of adolescents, marijuana users showed increased recruitment of executive attention regions during NoGo trials relative to baseline in a Go-NoGo task (Tapert et al., 2008).

Dysfunction in executive attention neuro-circuitry may be related to increased amygdala responsiveness to threat in patients with substance abuse. Executive attention neuro-circuitry involves the dlPFC and parietal cortices and allows the priming of task-relevant representations at the expense of irrelevant ones (Desimone and Duncan, 1995). This increased priming of task-relevant stimuli inhibits the representation of emotional distractors and results in reduced amygdala responses to these distractors (Blair et al., 2007). Executive attention can be recruited explicitly within cognitive reappraisal emotion regulation paradigms (Ochsner and Gross, 2005) but also implicitly through emotion distraction paradigms (Erthal et al., 2005). Both executive attention and emotional responsiveness systems are implicated in exteroception, or processing self-relevant external stimuli, and is thought to play a role in the development and maintenance of substance abuse (DeWitt et al., 2015). If alcohol and/or cannabis abuse compromise executive attention, then representation of external task-relevant stimuli should be impaired, resulting in compromised emotion regulation and increased emotional responsiveness. Alternatively, alcohol and/or cannabis abuse may compromise neural systems underlying exteroception relatively independently, resulting in reduced representation of task-relevant stimuli regardless of emotional stimuli and/or increased emotional responsiveness regardless of task demands.

In the current study, we implemented an emotion distraction task, the Affective Stroop task (aST; Blair et al., 2007) in adolescents showing varying levels of AUD and CUD symptomatology. In the aST, participants are instructed to determine the quantity of numbers displayed on the screen that are temporally bracketed by either emotional or neutral distracters. Work with healthy adolescents (Hwang et al., 2014) and adults (Blair et al., 2007) reveals that task performance is associated with decreased amygdala responsiveness to emotional distracters and increased recruitment of regions mediating behavioral inhibition (ACC, dmPFC, aIC, and iFG) and executive attention (dlPFC and parietal cortices) to task-relevant stimuli. The aST has been extensively used in work with both adolescent and adult clinical populations (Blair et al., 2013, Blair et al., 2012; Hwang et al., 2016, Hwang et al., 2015; White et al., 2014). Specifically, adults with GAD, SAD, and PTSD show compromised recruitment of ACC and/or parietal cortices during task relative to view trials (Blair et al., 2013, Blair et al., 2012) while adolescents with ADHD, show reduced dmPFC activity during incongruent trials relative to typically developing (TD) adolescents (Hwang et al., 2015). Furthermore, in adolescents with disruptive behavior disorders (DBDs), there is decreased recruitment of aIC in incongruent relative to view trials and the degree to which this is compromised relates to impulsivity symptoms within this sample (Hwang et al., 2016). In addition, adolescents with DBDs and high levels of callous-unemotional traits showed reduced vmPFC and amygdala responsiveness to negatively valenced stimuli (Hwang et al., 2016). In short, the aST has been successfully used to show dysfunction in emotion processing, behavioral inhibition, and executive attention neuro-circuitries in adult and adolescent clinical populations.

We hypothesized that: (i) participants with high levels of AUD and CUD symptoms would show increased recruitment of the region implicated in emotional responding to both positively and negatively valenced stimuli (amygdala); and (ii) participants with at least high levels of AUD symptomatology would show reduced recruitment of regions implicated in behavioral inhibition (dmPFC/ACC and/or aIC/iFG) and/or executive (dlPFC and/or parietal cortices) to task relative to view trials.

## 2 Materials and methods

### 2.1 Participants

Study participants included 96 youths aged 14–18 years from both a residential treatment facility and the community. 14 participants were excluded due to excessive movement (>10% volumes censored at >1 mm motion across adjacent volumes) or low accuracy on the task (<60% accuracy; average AUDIT of excluded participants = 4.2, average CUDIT of excluded participants = 5.0). This resulted in a final sample of 82 youths (47 youths from the residential treatment facility and 35 from the community); average age = 16.1 (SD = 1.32), IQ = 100.6 (SD = 10.13) and 51 male. Clinical characterization was done through psychiatric interviews by licensed and board-certified psychiatrists with the participants and their parents. Youths with significant substance abuse histories were residents of the residential treatment facility and were abstinent for at least four weeks prior to scanning.

49 youths endorsed having used, and 33 youths denied having used, alcohol and/or cannabis on the Alcohol Use Disorder Identification Test (AUDIT) and the Cannabis Use Disorder Identification Test (CUDIT), respectively (Adamson et al., 2010; Fairlie et al., 2006; Saunders et al., 1993). The range of AUDIT scores and CUDIT scores was 0–22 (M = 2.9; SD = 4.65) and 0–32 (M = 7.0; SD = 8.96), respectively. AUDIT scores, but not CUDIT scores, were significantly related with age [AUDIT: *r* = 0.26, *p* = 0.02; CUDIT: *r* = 0.19, *ns*] while neither AUDIT nor CUDIT scores were significantly related to IQ [AUDIT: *r* = −0.118, *ns*; CUDIT: *r* = −0.159, *ns*]. There were no differences in AUDIT or CUDIT scores between males and females [AUDIT: *t*(80) = −0.76, *ns*; CUDIT: *t*(80) = 1.09, *ns*].

Of the youths endorsing alcohol and/or cannabis use during their lifetimes, 14 youths showed subclinical levels of alcohol and/or cannabis use while 35 met the clinical cutoffs on the AUDIT and/or CUDIT suggestive of adolescent AUD (AUDIT score ≥ 4) or CUD (CUDIT score ≥ 8), respectively (Adamson et al., 2010; Fairlie et al., 2006). 21 participants had an AUDIT score ≥ 4 and 29 participants had a CUDIT score ≥ 8. In line with previous work indicating the high comorbidity of AUD and CUD (Mason et al., 2013; Moss et al., 2014), 15 participants had both an AUDIT score ≥ 4 and CUDIT score ≥ 8.

Exclusion criteria included pervasive developmental disorder, Tourette's syndrome, lifetime history of psychosis, neurological disorder, head trauma, and non-psychiatric medical illnesses requiring medications that may have psychotropic effects (e.g. beta-blockers, steroids), and IQ < 75. The Institutional Review Board at Boys Town National Research Hospital approved the study procedures and informed assent/consent was obtained from all participants and their parents or legal guardians.

### 2.2 Measures

#### 2.2.1 Affective Stroop task (aST)

**Fig. 1:**
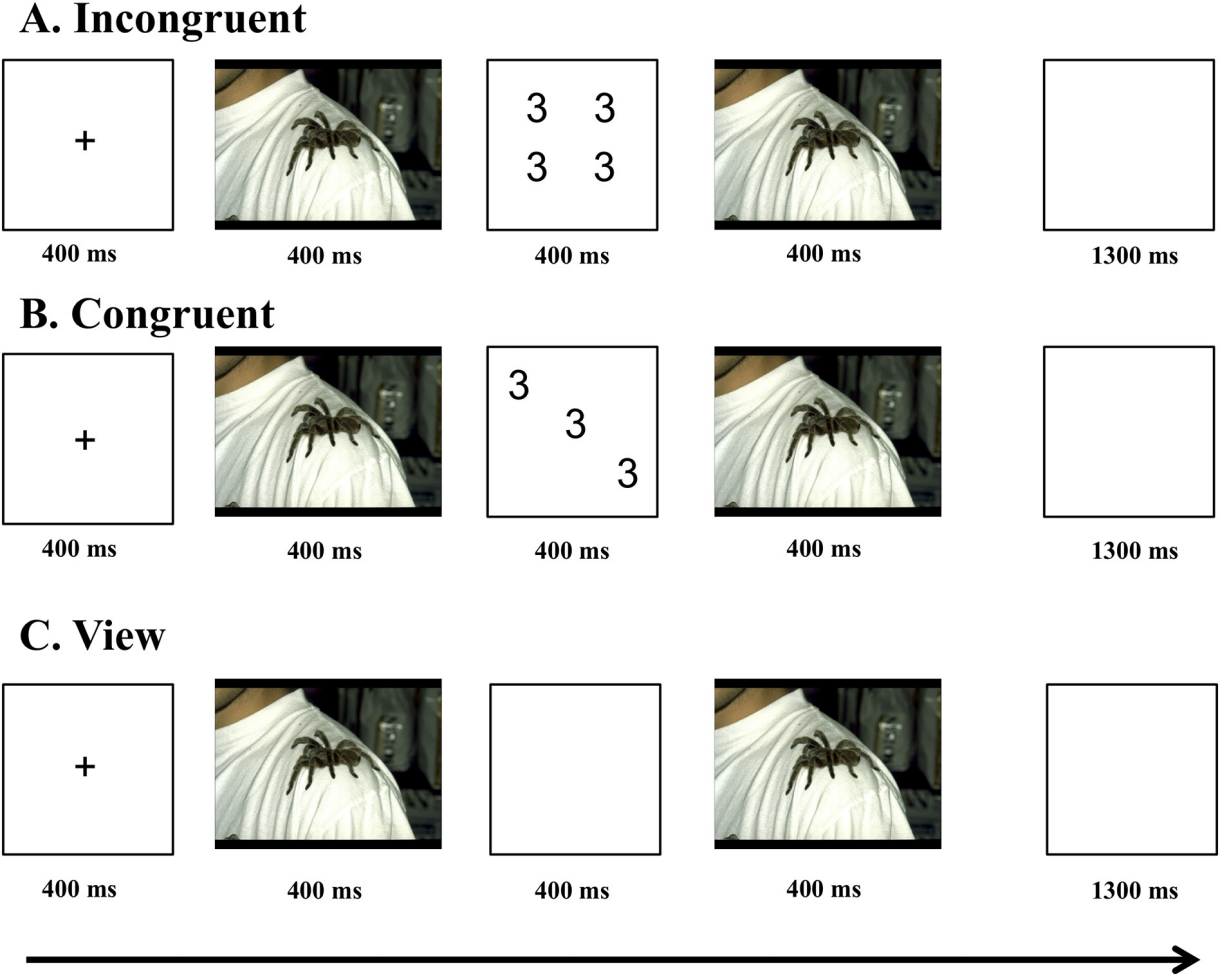
Diagram of aST for a trial with a negatively valenced stimulus. The (A) first row indicates an incongruent trial, the (B) second row indicates a congruent trial, and the (C) third row indicates a view trial.

An adapted version of the Affective Stroop task (Blair et al., 2007) was administered during fMRI scanning (see Fig. 1). The emotional stimuli consisted of 16 negative, 16 neutral, and 16 positive pictures selected from the International Affective Picture System (IAPS; Lang et al., 1988). The mean valence and arousal values on a 9-point scale, respectively, were 3.2 (SD = 0.71) and 1.7 (SD = 0.28) for negative images; 4.9 (SD = 0.30) and 1.1 (SD = 0.22) for neutral images; and 7.4 (SD = 0.47) and 1.61 (SD = 0.31) for positive images. The individual cognitive task stimuli consisted of displays of numbers and the cognitive task involved deciding how many numbers were displayed in each display (see Fig. 1 for example stimuli). Specifically, subjects pressed button 3, 4, 5, or 6 to indicate whether there were 3, 4, 5, or 6 numbers in the display.

Each trial began with a fixation point presented in the middle of the screen. For the number trials, the fixation point was replaced by the first picture stimuli presented for 400 ms, followed by the numerical display presented for 400 ms, followed by the second picture display presented for 400 ms, followed by a blank stimulus for 1300 (see Fig. 1). On incongruent trials, the Arabic numeral distracter information was inconsistent with the numerosity information (e.g., four 3s; Fig. 1A). On congruent trials, the Arabic numeral distracter information was consistent with the numerosity information; (e.g., three 3s; Fig. 1B). For view trials, there was no numerical display; the numerical display was replaced by a fixation point (see Fig. 1C). Participants completed two identical runs of the task. In each run, each subject was presented with 16 trials of each of the 9 emotion-by-task conditions. This resulted in 288 total trials. In addition, 40 fixation points (staying on the screen for the duration of a condition trial 2500 ms) were randomly presented throughout each run in order to create a baseline. Thus, overall each subject was presented with 32 trials of each of the 9 emotion-by-task conditions resulting in 288 total trials.

#### 2.2.2 Substance use disorder assessments

Youths completed the Alcohol Use Disorder Identification Test (AUDIT) and Cannabis Use Disorder Identification Test (CUDIT). These scales assess overall alcohol/cannabis consumption over the past year as well as symptoms of alcohol/cannabis abuse and dependence. These scales show high validity, as elevated scores on these scales indicate a high probability of a AUD and/or CUD diagnosis (Adamson et al., 2010; Fairlie et al., 2006; Saunders et al., 1993). Smoking status was determined using the Monitoring the Future Survey (Miech et al., 2016). As can be seen in Table 1, participants meeting clinical cut-offs on the AUDIT/CUDIT endorsed regular past smoking while sub-clinical levels of AUDIT/CUDIT symptomatology were associated with rare past usage. Most participants with no AUDIT/CUDIT symptomatology endorsed no prior smoking history.

**Table 1:** Clinical and demographic characteristics.

	No SU (n = 33)	Subclinical SU (n = 14)	AUDIT ≥ 4 (n = 21)^a^	CUDIT ≥ 8 (n = 29)^a^
Age	15.6 (1.37)	16.6 (1.34)	16.5 (1.17)	16.2 (1.20)
IQ	100.8 (9.36)	103.4 (10.83)	99.7 (11.67)	98.6 (11.39)
% male	63.6%	35.7%	57.1%	75.9%
ADHD	36.3%	28.6%	61.9%	68.9%
CD	24.2%	50.0%	66.7%	75.9%
PTSD	18.1%	7.1%	28.6%	17.2%
SAD	15.1%	14.3%	38.1%	27.6%
GAD	15.1%	7.1%	52.4%	44.8%
MDD	18.2%	28.6%	38.1%	24.1%
CBCL ADHD raw score	3.5 (3.81)	4.6 (4.31)	6.1 (3.56)	6.6 (2.81)
CBCL CD raw score	5.9 (8.69)	8.9 (8.25)	12.3 (7.69)	12.3 (6.47)
CBCL Externalizing T-score	52.8 (16.93)	59.9 (17.58)	68.1 (12.89)	69.1 (8.27)
SCARED Social Anxiety score	4.8 (3.36)	4.3 (3.43)	6.1 (4.71)	5.5 (4.16)
SCARED Generalized Anxiety score	5.3 (4.31)	5.0 (3.44)	9.0 (5.65)	7.3 (5.27)
SCARED Total score	18.3 (13.98)	14.3 (8.71)	28.2 (20.19)	21.9 (15.99)
MFQ	9.3 (11.90)	10.1 (9.69)	19.1 (17.33)	13.1 (12.12)
AUDIT	0 (0)	1.4 (1.15)	9.1 (5.36)	5.6 (5.43)
CUDIT	0 (0)	3.1 (2.81)	13.9 (8.89)	17.7 (6.43)
Smoking	0.2 (0.65)	1.4 (1.15)	2.8 (1.33)	2.7 (1.40)

Note: ADHD = Attention Deficit/Hyperactivity Disorder; CD = Conduct Disorder; PTSD = Post Traumatic Stress Disorder; SAD = Social Anxiety Disorder; GAD = Generalized Anxiety Disorder; MDD = Major Depressive Disorder; diagnoses may overlap.

a 15 participants had an AUDIT score ≥ 4 and a CUDIT score ≥ 8.

#### 2.2.3 Psychiatric symptomatology assessments

In order to provide more details on psychiatric co-morbidities, levels of externalizing, anxiety, and depressive symptomatology were assessed. The externalizing problems subscale of the parent-report version of the Childhood Behavior Checklist (CBCL) was used to assess externalizing behaviors (Achenbach and Rescorla, 2001). The self-report version of the Screen for Child Anxiety and Related Disorders (SCARED) was used to assess levels of anxiety symptoms (Birmaher et al., 1997). The self-report version of the Mood and Feelings Questionnaire (MFQ) was used to assess levels of depressive symptoms (Angold et al., 1995).

### 2.3 Scanning parameters

Whole-brain blood oxygen level dependent (BOLD) data were acquired using a 3.0 Tesla Siemens Skyra Magnetic Resonance Scanner. A total of 384 functional images were taken, divided over two runs, with a T2* weighted gradient echo planar imaging (EPI) sequence (repetition time = 2500 ms; echo time = 27 ms, 94 × 94 matrix; 90° flip angle; 240 mm field of view). Whole-brain coverage was obtained with 43 axial slices (thickness, 2.5 mm; voxel size 2.6 × 2.6 × 2.5 mm^3^). In the same session, a high-resolution T1 anatomical scan (MP-RAGE, repetition time = 2200 ms, echo time = 2.48 ms; 230 mm field of view; 8° flip angle; 256 × 208 matrix) was acquired in register with the EPI dataset. Whole-brain coverage was obtained with 176 axial slices (thickness, 1 mm; voxel size 0.9 × 0.9 × 1 mm^3^).

### 2.4 fMRI analysis: data preprocessing and individual level analysis

Functional MRI data were preprocessed and analyzed using Analysis of Functional NeuroImages (AFNI) software (Cox, 1996). The first four volumes in each scan were discarded. The anatomical scan for each participant was registered to the Talairach and Tournoux atlas (Talairach and Tournoux, 1988) using the TT_N27 template and each participant's functional EPI data were registered to their Talairach anatomical scan in AFNI. Functional images were motion corrected and spatially smoothed with a 6-mm full width half maximum Gaussian kernel. The data then underwent time series normalization by dividing the signal intensity of a voxel at each time-point by the mean signal intensity of that voxel for each run and multiplying by 100. Therefore, the resultant regression coefficients are representative of a percentage of signal change from the mean.

Afterward, regressors were generated by convolving the train of stimulus events with a gamma variate hemodynamic response function to account for the slow hemodynamic response. The ten regressors were: (i) positive images, incongruent numerosity; (ii) positive images, congruent numerosity; (iii) positive images, view; (iv) neutral images, incongruent numerosity; (v) neutral images, congruent numerosity; (vi) neutral images, view; (vii) negative images, incongruent numerosity; (viii) negative images, congruent numerosity; (ix) negative images, view; (x) missed/incorrect responses. GLM fitting was performed with these ten regressors, six regressors modeling motion, and a regressor modeling a first-order baseline drift function. This produced a β coefficient and an associated *t*-statistic for each voxel and regressor.

### 2.5 fMRI analysis: group analysis

To reduce skewness and kurtosis, a Blom Transformation was applied to the participants' AUDIT and CUDIT scores. This is a normalization procedure which ranks orders, and then standardizes values within a dataset (Blom, 1958). The pre-transformation skewness values for AUDIT and CUDIT scores were 2.4 and 1.1, respectively. Post-transformation, the skewness values were 0.8 and 0.7, respectively. The pre-transformation kurtosis values for AUDIT and CUDIT scores were 6.4 and 0.3, respectively. Post-transformation, the kurtosis values were −0.4 and −0.6, respectively. The Blom-Transformed standardized AUDIT and CUDIT scores were used for all analyses. For the group-level analyses, a 3 (Emotion: Positive, Neutral, Negative) × 3 (Task Condition: Incongruent, Congruent, View) repeated measures ANCOVA with AUDIT and CUDIT scores as continuous covariates was performed on the BOLD data within a grey matter mask created in AFNI. Follow-up testing was performed within SPSS 22.0 and freely available online tools (Lee and Preacher, 2013). For significant AUDIT-by-emotion interactions, Steiger Z-tests were used to compare the partial correlations between AUDIT scores and BOLD responses (controlling for CUDIT scores and AUDIT-by-CUDIT interactions) in the positive trials, neutral trials, and negative trials (Steiger, 1980). A similar procedure was used for any significant CUDIT-by-emotion, AUDIT-by-task condition, and CUDIT-by-task condition interactions. For four-way interactions, a bootstrapping procedure using the PROCESS macro for SPSS (Preacher and Hayes, 2004) was used to examine how CUD symptomatology moderated the effect of AUD symptomatology on BOLD response within each of the 9 emotion-by-task condition trial types. For these follow-up tests, the AUDIT-by-CUDIT interaction term was considered significant at a threshold of *p* = 0.05, Bonferroni corrected. For each trial type that was identified as significant, the Johnson-Neyman technique was used to investigate heterogeneity of the relationship between AUDIT scores and BOLD responses at different levels of CUDIT scores (Kowalski et al., 1994). The Johnson-Neyman technique identifies specific regions of interest within the distribution of CUDIT scores where the relationship between AUDIT scores and BOLD responses was significant. The Johnson-Neyman technique was used to probe these interactions because it provides information regarding the nature of the relationship between AUDIT scores and BOLD responses across the entire distribution of CUDIT scores (Kowalski et al., 1994). To facilitate future meta-analytic work, effect sizes (Partial η^2^) for all clusters are reported.

The AFNI 3dClustSim program, using the autocorrelation function (-acf), was used to establish a family-wise error correction for multiple comparisons for the amygdala ROI and whole-brain analysis (Cox et al., 2017). Spatial autocorrelation was estimated from residuals from the individual-level GLMs. Given our a priori hypotheses regarding the amygdala, regions of interest (ROIs) for left and right amygdala were specified as anatomically defined masks (Eickhoff-Zilles Architectonic Atlas 50% probability mask; Amunts et al., 2005). This yielded a threshold of 5 voxels at an initial threshold of *p* = 0.02 for the amygdala ROI. The whole-brain analysis yielded a threshold of 19 voxels at an initial threshold of *p* = 0.001. Post-hoc analyses were conducted on the percent signal change taken from all significant voxels within each ROI and whole-brain functional masks generated by AFNI to examine significant main effects and interactions with planned follow-up testing within SPSS 22.0 (IBM SPSS Statistics For MacOSX, 2012).

## 3 Results

### 3.1 Clinical relationships

Correlation analyses were conducted to determine the relationships between AUD, CUD and psychiatric symptom levels dimensionally. These revealed positive correlations between AUDIT and CUDIT scores [*r* = 0.63, *p* < 0.001] and AUDIT scores and levels of externalizing problems (CBCL externalizing T-score), anxiety (SCARED total score) and depressive symptoms (MFQ) [*r*'s = 0.27–0.33, *p*'s < 0.05]. CUDIT scores were positively correlated with level of externalizing problems [*r* = 0.39, *p* = 0.001], and with level of anxiety symptoms and level of depressive symptoms at trend levels [*r*'s = 0.19–0.192, *p*'s < 0.10]. Additionally, both AUDIT scores and CUDIT scores were positively related to level of smoking [*r*'s = 0.65 and 0.70, respectively; p's < 0.001]. Importantly, there were no differential correlations between AUDIT and CUDIT scores and levels of externalizing problems, anxiety symptoms, or depressive symptoms [Steiger's Z's = 0.70–1.18, *ns*]. There were also no differential correlations between AUDIT and CUDIT scores and level of smoking [Steiger Z = 0.71, *ns*]. Ten participants had missing data for the CBCL, six had missing data for the SCARED, and two each had missing MFQ and smoking data. There were no differences in AUDIT scores or CUDIT scores between participants who were missing data on the CBCL, SCARED, MFQ, and/or smoking data and those who were not missing these data [t's < 1.36, *ns*]. See Table 1, Table 2 for more details.

**Table 2:** Zero-order correlations across demographic and clinical variables.

	1	2	3	4	5	6	7	8	9	10	11	12	13
1. Age													
2. IQ	0.19												
3. Gender^a^	0.07	0.04											
4. AUDIT	0.26⁎	−0.12	−0.09										
5. CUDIT	0.19	−0.16	0.12	0.63†									
6. Smoking	0.21	−0.05	0.10	0.70†	0.65†								
7. CBCL - ADHD	−0.08	−0.21	0.20	0.19	0.31†	0.37†							
8. CBCL - Conduct	−0.11	−0.24⁎	0.18	0.26⁎	0.30⁎	0.38†	0.77†						
9. CBCL - Externalizing	−0.11	−0.32†	0.21	0.33†	0.39†	0.43†	0.83†	0.92†					
10. SCARED - SAD	0.14	0.15	−0.23⁎	0.16	0.15	0.09	0.02	−0.16	−0.12				
11. SCARED - GAD	0.09	−0.2	−0.34†	0.32†	0.23⁎	0.17	0.06	0.00	0.06	0.63†			
12. SCARED - Total	0.02	−0.03	−0.32†	0.27⁎	0.19	0.11	0.13	0.02	0.10	0.77†	0.89†		
13. MFQ - Total	−0.11	−0.12	−0.31†	0.31†	0.19	0.06	0.16	0.22	0.25⁎	0.44†	0.60†	0.70†	

a Gender coded as female = 0, male = 1.

⁎ Significant at *p* < 0.05.

† Significant at *p* < 0.01.

### 3.2 Behavioral results

Two 3 (Emotion: Positive, Neutral, Negative) × 2 (Task Condition: Incongruent, Congruent) repeated measures ANCOVAs using the normalized Blom-Transformed AUDIT and CUDIT scores as continuous covariates were conducted on the aST accuracy and reaction time (RT) data. Accuracy on the aST ranged from 60% to 99%. There was a main effect of task condition, [*F*(1,78) = 33.49, *p* < 0.001]; participants were less accurate on incongruent trials [*M* = 81.33%, SD = 14.61%] relative to congruent trials [*M* = 86.93%, SD = 10.04%]. The emotion main effect, covariate-by-emotion interaction effects, and covariate-by-task condition interaction effects were not significant.

With respect to RT, there was again a main effect of task condition [*F*(1,78) = 167.33, *p* < 0.001]; participants responded slower on incongruent trials [*M* = 854.96, SD = 200.89] than congruent trials [*M* = 787.52, SD = 207.56]. The emotion main effect, covariate-by-emotion interaction effects, and covariate-by-task condition interaction effects were not significant.

### 3.3 Movement data

Fourteen participants were excluded due to excessive motion or low accuracy on the task. Within the final sample (N = 82), volumes were censored if there was >1 mm motion across adjacent volumes. No participant in the final sample for the current study had >5% censored volumes. There were no relationships between either AUDIT scores or CUDIT scores and censored volumes, average motion per volume, or maximum displacement during scanning within the final sample [*r*'s = −0.10–0.20, *ns*].

### 3.4 fMRI results

The goal of the current study was to examine whether level of adolescent AUD and CUD symptomatology was related to dysfunction in brain regions associated with emotional responding and executive attention. We ran a 3 (Emotion: Positive, Neutral, Negative) by 3 (Task Condition: Incongruent, Congruent, View) repeated measures ANCOVA with the Blom-Transformed standardized AUDIT and CUDIT scores as continuous covariates on the BOLD response data. This revealed regions showing AUDIT-by-emotion, AUDIT-by-task condition, CUDIT-by-task condition and AUDIT-by-CUDIT-by-emotion-by-task condition interactions. Regions showing main effects of emotion and task and emotion-by-tasks interaction are reported in the Supplemental material (Table S1). No regions showed significant AUDIT main effects, CUDIT main effects, or AUDIT-by-CUDIT interactions. No regions showed significant CUDIT-by-emotion, AUDIT-by-CUDIT-by-emotion, AUDIT-by-CUDIT-by-task condition, AUDIT-by-emotion-by-task condition, or CUDIT-by-emotion-by task condition interactions:

#### 3.4.1 Amygdala ROI

##### 3.4.1.1 AUDIT-by-emotion interaction

**Fig. 2:**
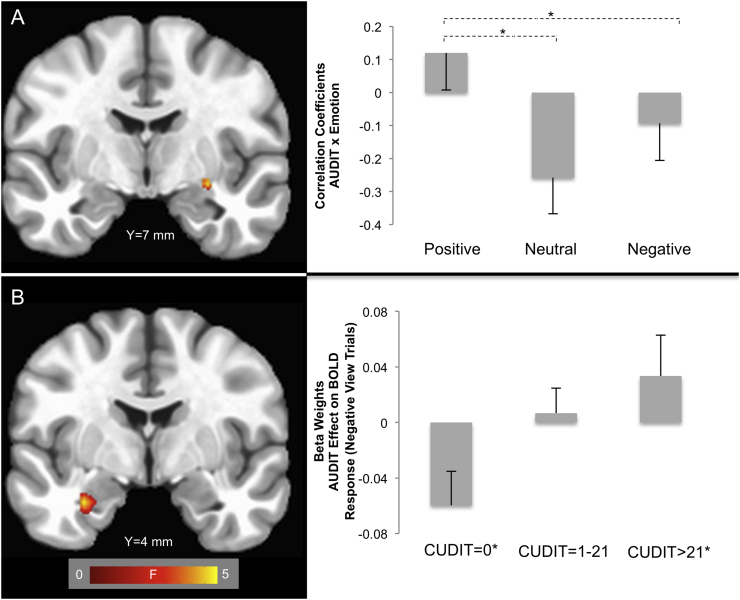
AUDIT-by-emotion interaction within the (A) Amygdala ROI (x = 29 mm, y = −7 mm, z = −7 mm). Participants with higher AUDIT scores showed increased responses to emotional relative to neutral stimuli (k = 5 voxels). Values in the bar graph represent the correlation coefficients between AUDIT scores and BOLD responses for each emotion; * indicates significant differences between partial correlation values (Steiger's Z > 1.96, *p* < 0.05). (B) AUDIT-by-CUDIT interaction effect within the negative view trials (k = 9 voxels). Values in the bar graph represent the beta weights for the effect of AUDIT score on BOLD response within the range of CUDIT scores indicated. * indicates regions of interest significant at *p* < 0.05 identified via the Johnson-Neyman technique.

There was a significant AUDIT-by-emotion interaction within the right amygdala (Fig. 2). With increasing AUDIT scores, there were increasing BOLD responses for positive relative to both neutral and negative stimuli [Steiger's Z's = 3.37 & 2.30, *p* < 0.001 & *p* < 0.05 respectively]. The ROI analysis revealed no significant CUDIT-by-emotion interactions. The AUDIT-by-CUDIT-by-Emotion interaction within this cluster was not significant, indicating that the relationship between AUDIT scores and BOLD responses was consistent across all CUDIT scores.

##### 3.4.1.2 AUDIT-by-CUDIT-by-emotion-by-task condition interaction

There was a four-way interaction in the left amygdala ROI (Fig. 2). Utilizing a bootstrapping procedure for the moderation analysis, it was found that there was a significant AUDIT-by-CUDIT interaction effect in negative view trials. Using the Johnson-Neyman technique, it was found that there was a negative relationship between AUDIT scores and activation on negative view trials at relatively low CUD symptomatology (CUDIT = 0). However, there was a positive relationship between AUDIT scores and activation on negative view trials at relatively high CUD symptomatology (CUDIT > 21).

#### 3.4.2 Whole-brain analysis

##### 3.4.2.1 AUDIT-by-task condition interaction

**Fig. 3:**
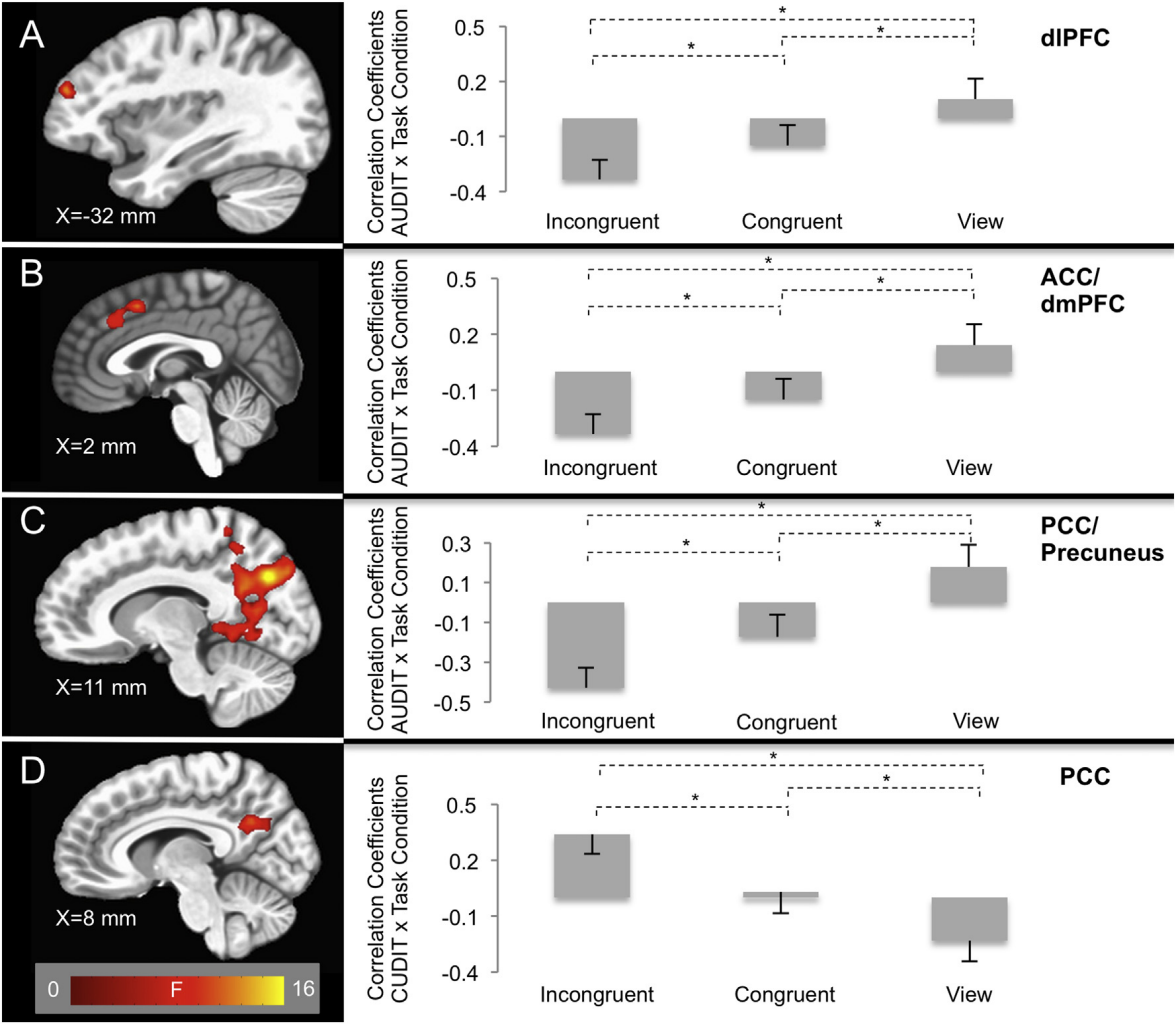
AUDIT-by-task condition interactions within the (A) dlPFC (x = 26 mm, y = 35 mm, z = 44 mm); (B) ACC/dmPFC (x = 2 mm, y = 11 mm, z = 44 mm); and (C) PCC/Precuneus (x = 11 mm, y = −67 mm, z = 29 mm). Participants with higher AUDIT scores showed decreased responses in these brain regions during incongruent trials relative to congruent and view trials. Values in the bar graphs represent the correlation coefficients between AUDIT scores and BOLD response for each task condition within each cluster. CUDIT Score-by-task condition interactions within the (D) PCC (x = 8 mm, y = −52 mm, z = 26 mm). Participants with higher CUDIT scores showed increased responses in these brain regions during incongruent trials relative to congruent and view. Values in the bar graphs represent the correlation coefficients between CUDIT scores and BOLD responses for each task condition within each cluster. * indicates significant differences between partial correlation values (Steiger's Z > 1.96, *p* < 0.05).

There were significant AUDIT-by-task condition interactions within regions including dlPFC, iFG, middle frontal gyrus (MFG), ACC, dmPFC, precuneus, and posterior cingulate cortex (PCC; Fig. 3, Table 3). These regions overlapped with regions involved in the main effect of task condition (Table S1). In all but one region of dlPFC, BOLD responses were greater to task relative to view trials. Additionally, within ACC and dmPFC, BOLD responses were also greater to incongruent relative to congruent trials. Within all of these regions, increased AUDIT scores were associated with decreased activation for incongruent relative to congruent [Steiger's Z's = −2.08–3.26, *p*'s < 0.05; except MFG: Steiger's Z = −1.49, *ns*] and view trials [Steiger's Z's = −3.19–5.43, *p*'s < 0.002] and also congruent relative to view trials [Steiger's Z's = −2.46–4.26, *p*'s < 0.02]. As can be seen in Fig. 3, these data reflected decreasing responses during incongruent trials as a function of increasing levels of AUD symptomatology. None of these clusters revealed a significant AUDIT-by-CUDIT-by-Task Condition interaction, indicating that the relationship between AUDIT scores and BOLD responses were consistent across the entire distribution of CUDIT scores.

**Table 3:** Brain regions demonstrating significant AUDIT-by-task condition, CUDIT-by-task condition, and significant AUDIT-by-CUDIT-by-emotion-by-task condition interactions.

Coordinates of peak activation^b^								
Region^a^	Hemisphere	BA	x	y	z	*F*	Partial η^2^	Voxels
*AUDIT-by-task condition*								
dlPFC	R	8	29	35	44	18.21	0.189	109
dlPFC	L	10	−34	47	20	12.65	0.140	23
dlPFC/iFG	R	9	53	5	29	11.05	0.124	21
MFG	R	6	20	20	56	13.14	0.144	28
ACC/dmPFC	R/L	6/32	2	11	44	15.30	0.164	72
Precuneus/PCC	R/L	7/31	11	−67	29	23.49	0.231	1500
PCC	R	31	5	−31	47	10.99	0.123	28
iPL	R	40	35	−49	41	15.40	0.165	36
iPL	R	13/40	50	−43	23	14.02	0.152	26
iPL	R	40	50	−37	35	15.55	0.166	21
Postcentral gyrus	R	41	53	−19	14	11.57	0.129	25
Middle temporal gyrus	R	19	44	−61	11	17.10	0.180	77
Parahippocampal gyrus	L	27	−25	−34	−1	18.13	0.189	23

*CUDIT-by-task condition*								
PCC	R	31	11	−52	26	14.01	0.152	83
Precuneus	R	7/31	14	−70	29	12.00	0.133	29
Precuneus	L	31	−16	−67	26	12.31	0.136	26
iPL	R	39	35	−58	38	12.48	0.138	26
Middle temporal gyrus	R	19	44	−61	11	12.39	0.137	23
Middle temporal gyrus	R	39	50	−67	26	10.87	0.122	21
Culmen	L	–	−7	−61	−7	13.48	0.147	36
Cerebellum	L	–	−31	−67	−34	13.22	0.145	21
Cerebellum	L	–	−7	−82	−28	15.16	0.163	19

*AUDIT-by-CUDIT-by-emotion-by-task condition*								
iFG	L	9	−40	5	29	7.25	0.085	31

BA = Brodmann's Area.

a According to the Talairach Daemon Atlas (http://www.nitrc.org/projects/tal-daemon/).

b Based on the Tournoux & Talairach standard brain template.

##### 3.4.2.2 CUDIT-by-task condition interaction

There were significant CUDIT-by-task condition interactions within regions including PCC, precuneus, and inferior parietal lobule (iPL; Fig. 3, Table 3). These regions overlapped with regions involved in the main effect of task condition (Table S1). Within all of these regions, BOLD responses were greater to task relative to view trials. In these regions, as CUDIT scores increased, there was *increased* activation for incongruent relative to congruent [Steiger's Z's = 2.84–3.98, *p*'s < 0.005] and view trials [Steiger's Z's = 3.25–4.54, *p*'s < 0.001]. As can be seen in Fig. 3, these data reflected both increasing responses during incongruent trials and decreasing responses during view trials as a function of increasing levels of CUD symptomatology. None of these clusters revealed a significant AUDIT-by-CUDIT-by-Task Condition interaction, indicating that the relationship between AUDIT scores and BOLD responses were consistent across the entire distribution of CUDIT scores.

##### 3.4.2.3 AUDIT-by-CUDIT-by-emotion-by-task condition interaction

There was a significant four-way interaction effect in the left iFG (Table 3). Notably, a bootstrapping procedure for the moderation analysis revealed a significant AUDIT-by-CUDIT interaction effect for negative view trials; AUDIT scores were negatively associated with activation at lower CUD symptom levels (CUDIT < 4) but positively associated at high CUD symptom levels (CUDIT > 27).

### 3.5 Potential confounds

Since age was related to AUDIT scores, the same analysis was repeated with age as a covariate (Table S2). In addition, calculation of Mahalanobis Distance for each participant revealed four multivariate outliers within the dataset. Therefore, the same analysis was repeated with these participants removed from the dataset (Table S3). Since there is evidence that males and females may be differentially affected by alcohol and cannabis (Caldwell et al., 2005; Ketcherside et al., 2016; Peters et al., 2015), the same analysis was repeated with gender was entered as a covariate (Table S4). To rule out the possibility that smoking may have influenced our results, the same analysis was repeated with participants who endorsed current smoking excluded (Table S5). To rule out the possibility that over-representation of 0 for AUDIT and CUDIT scores biased our results, we re-ran the analysis in only individuals who reported alcohol and/or cannabis use (Table S6). All of these analyses yielded similar results (see Supplementary results; Tables S2–S6).

## 4 Discussion

This study examined the relationships between AUD and CUD severity and dysfunction in emotional and executive attention neuro-circuitry in adolescents. There were three main findings. First, increasing AUD, but not CUD, severity was associated with increasing amygdala responses to emotional relative to neutral stimuli. Second, increasing AUD severity was associated with decreasing levels of recruitment of regions implicated in executive attention for task relative to view trials. Third, increasing CUD severity was associated with increasing BOLD responses within PCC, precuneus, and iPL during task relative to view trials.

In line with our predictions, increasing severity of AUD symptomatology was associated with increasing amygdala responsiveness to emotional relative to neutral stimuli. This was seen for increasing AUD severity when responding to negative view trials if CUD symptomatology was high and for positive trials irrespective of task condition or level of CUD symptomatology. Previous work has suggested that chronic alcohol use leads to an increased stress response and hyper-responsiveness of the amygdala to threat stimuli (Koob and Volkow, 2016; Volkow et al., 2016). Thus, previous fMRI work has revealed that adults with alcohol dependence show increased amygdala responses to threat (Gilman and Hommer, 2008) and that increased amygdala threat responsiveness is a risk factor for the development of alcohol abuse in college students – at least for those showing reward hyporesponsiveness (Nikolova et al., 2016). However, no previous work has investigated amygdala responsiveness to threat or (non-alcohol cue) positive stimuli in adolescents with alcohol abuse histories. The current data complements the earlier work by indicating threat hyper-responsiveness in adolescents as a function of AUD severity (at least for those with relatively high levels of CUD) and extends this earlier work by indicating elevated responsiveness to positive stimuli as a function of AUD severity also. The AUDIT-by-Emotion amygdala interaction is right lateralized. Lateralized amygdala findings are not uncommon in the literature though their interpretation remains speculative. A meta-analytic review of the data found evidence of a relative left amygdala lateralization for stimuli containing language and a relative right-lateralization for masked stimuli (Costafreda et al., 2008). This prompted the suggestion that the right amygdala might play a greater role in initial stimulus detection (Costafreda et al., 2008). On this basis, it could be suggested that adolescents with high levels of AUD symptoms are particularly responsive to the initial detection of emotional stimuli. However, this speculation goes considerably beyond the data.

It should be noted that severity of CUD symptomatology was *not* related to amygdala responsiveness to emotional stimuli. If this result replicates, models assuming that substance abuse generally leads to increased amygdala responsiveness (Koob and Volkow, 2016; Volkow et al., 2016) may need adjustment for adolescent substance use. The current data imply that correlates of AUD symptomatology differ from those of CUD symptomatology in adolescents and that it is only alcohol abuse that leads to exaggerated amygdala responsiveness. The current findings are inconsistent with those of Spechler et al. (2015) who reported that adolescents with cannabis use histories show increased amygdala sensitivity to angry faces (Spechler et al., 2015). However, the Spechler et al. study involved adolescents who mostly reported very low levels of cannabis use (49/70 cannabis users endorsed only using marijuana once or twice in their lives). Moreover, the current study differed from that of Spechler et al. with respect to psychiatric co-morbidity. It could be argued that the psychiatric co-morbidity camouflaged any relationship between CUD symptomatology and amygdala responsiveness. However, it should be noted that this was not the case with respect to AUD symptomatology and amygdala responsiveness (yet psychiatric co-morbidity was comparably related to AUDIT scores as CUDIT scores).

In line with predictions, increasing severity of AUD symptomatology was associated with reduced recruitment of ACC/dmPFC and iFG for incongruent relative to both congruent and view trials. Both ACC/dmPFC and iFG have been implicated in behavioral inhibition (Criaud and Boulinguez, 2013). Moreover, animal work has suggested that adolescent alcohol use is related to disrupted prefrontal cortex development and deficits in response inhibition during adulthood (Gass et al., 2014; Irimia et al., 2015; Spear, 2016) while human neuro-psychological work has revealed impairment on measures of behavioral inhibition in adults with AUD (Czapla et al., 2016). The current data suggest that increasing levels of alcohol abuse are associated, even in adolescence, with compromised recruitments of regions implicated in behavioral inhibition (even though increasing AUDIT scores were not related to behavioral performance on the current task).

Additionally, the ACC/dmPFC contains dense projections to and from brain regions involved in executive attention, such as dlPFC and iPL (Desimone and Duncan, 1995). The current study showed that increasing AUD symptomatology was associated with decreasing recruitment of dlPFC and iPL for incongruent relative to congruent and congruent relative to view trials. This is consistent with prior work showing reduced activity in these brain regions in adults (Ahmadi et al., 2013; Claus et al., 2013; Li et al., 2009) and youths (Thayer et al., 2015) with alcohol use histories. Notably, though, within the context of the aST, activity in these brain regions is thought to reflect a putative role in priming task-relevant stimuli and consequent decreased representation of and responsiveness to emotional stimuli; i.e., emotional regulation (Blair et al., 2007). In short, the findings of a negative relationship between response inhibition and executive attention neuro-circuitries and AUD symptoms, when combined with the positive relationship between amygdala responsiveness to emotional stimuli and AUD symptoms, might at least partly reflect the compromised functioning of this form of emotional regulation. It should be noted, however, that there was no evidence of any AUDIT-by-Emotion-by-Task Condition interactions; i.e., there were no indications of a failure to reduce emotional responsiveness as a function of AUD severity during negative task trials. Instead, AUD severity was associated with increased responsiveness across emotion conditions and might be particularly increased in negative view trials within IFG. As such, we assume that AUD severity is associated with increased emotional responsiveness that is independent of any failure in executive attention mediated emotional regulation.

Our third main finding was that increasing CUDIT scores were related to increasing activity within PCC, precuneus, and iPL during task relative to view trials. There have been suggestions that substance abuse, particularly cannabis abuse, may lead to increased prefrontal inefficiency; i.e., substance abuse may lead to compromised functioning of specific regions such that these regions need to be activated more strongly in order to produce successful task performance (Gruber et al., 2013; Luijten et al., 2014; Tapert et al., 2008). There have also been suggestions that patients with other psychiatric conditions also show indications of prefrontal inefficiency (Mitterschiffthaler et al., 2008; Wagner et al., 2006). In the current study, we saw clear indications of disruption in the functioning of frontal cortex particularly as a result of severity of AUD. However, increasing severity of AUD was associated with *decreasing* responsiveness within regions including dlPFC, iFG, middle frontal gyrus (MFG), ACC and dmPFC; i.e., increasing AUD severity was associated with a decreased ability to recruit these regions rather than revealing increasing, compensatory activity. In contrast, increasing CUD symptom severity *was* associated with increasing responsiveness within PCC, precuneus, and inferior parietal lobule in response to task trials; all regions implicated in responding to task trials. As such, these data might indicate a form of posterior attentional system inefficiency relating to CUD severity. Increasing CUD severity may have required participants to show stronger activation of these regions for successful task performance. While a compensatory account might explain the data of the current study, it is unclear why higher levels of CUD symptomatology would be associated with compensation while higher levels of AUD symptomatology would be associated with disrupted functioning (particularly when neither level of symptomatology related to behavioral performance). Alternatively, increased activity in the PCC and precuneus could reflect a failure of the default mode network to fully deactivate during task trials. This might reflect differences in concentration as a function of substance use and task difficulty (anonymous reviewer's suggestion). However, only these regions within the default mode network showed this effect. Even at more lenient thresholds (initial *p* = 0.005, k = 10 voxels), no significant clusters emerged within other regions implicated in the default mode network. It is unclear why differences in concentration would have selective effects within the default mode network.

The results of this study must be viewed in light of five caveats. First, we did not conduct urine or Breathalyzer testing for substance use at the time of scanning. However, youths with significant substance abuse histories were residents of a highly supervised residential treatment facility and had been abstinent for at least four weeks prior to scanning, mitigating this concern. Another significant caveat is that this study was cross-sectional in nature. As such, it is not possible to be certain whether the observed relationships between levels of AUD and CUD symptomatology and brain function reflected impact of substance abuse on the developing brain or pre-existing risk factors for the emergence of symptomatology. Animal and longitudinal neuroimaging work has shown that alcohol and cannabis use alter neurodevelopment (Spear, 2016; Squeglia et al., 2015). However, dysfunction in behavioral inhibition/top-down attention systems is also predictive of later problematic substance use (Norman et al., 2011). One reason to believe that the current results are more reflective of the impact of AUD/CUD on the developing brain is that there were differential relationships between AUD and CUD symptomatology on brain function. It is not clear that there are pre-existing neural risk factors that place the individual at risk specifically for AUD rather than CUD. However, future longitudinal work would need to confirm this suggestion. Third, the sample investigated here reflected clinical reality; i.e., there was a high degree of psychiatric co-morbidity in the participants that was particularly marked in those participants scoring high on the AUDIT/CUDIT. As such, the findings presented here might reflect psychopathology related to the co-morbid conditions rather than AUD/CUD symptomatology. It would be possible to test participants without co-morbid pathology. However, this would mean investigating a clinically atypical sample. Moreover, increasing substance abuse is hypothesized to compromise functions associated with the emergence of many of these psychiatric conditions (Koob and Volkow, 2016; Volkow et al., 2016). Critically though, and mitigating this concern, there were no significant differences between the relationships of externalizing, anxiety, or depressive psychopathologies and AUD relative to CUD severity. As such, it is unclear how psychiatric comorbidities could account for the current data. Fifth, other indices of substance involvement were not available (e.g., age of first use, cumulative exposure). Interestingly, using a Stop-Signal task, Filbey and Yezhuvath (2013) found that dependent, relative to non-dependent, marijuana using adults showed increased connectivity between right frontal cortex and substantia nigra/subthalamic nucleus and that the strength of this increased connectivity was modulated by both age of onset and quantity of cannabis use. In short, it is likely that these latter variables may modulate the strength of the findings here.

In summary, we found differential patterns of dysfunction associated with AUD and CUD symptomatologies. Elevated AUD symptomatology was associated with increased amygdala responses to positive relative to neutral stimuli and decreased responses in brain regions associated with behavioral inhibition and executive attention during incongruent relative to congruent trials. In contrast, elevated CUD symptomatology was associated with *increased* responses in the PCC, precuneus, and iPL for incongruent relative to congruent and view trials. These data suggest that correlates of AUD symptomatology differ from those of CUD symptomatology.

The following are the supplementary data related to this article.

Supplementary material Image 1

**Fig. S1:**
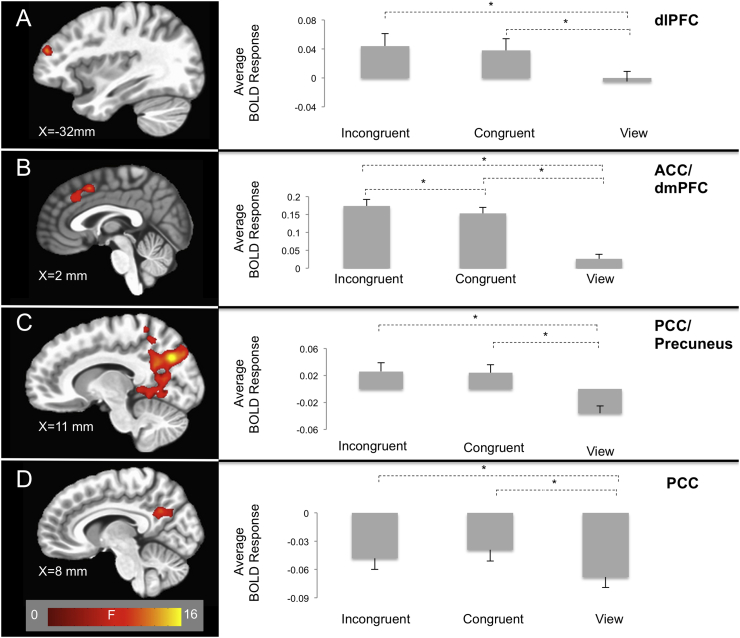
Main effects of task condition within clusters displayed in Fig. 3. * indicates significant differences at *p* < 0.05.

## References

[bb0005] Achenbach T.M., Rescorla L.A. (2001). Manual for the ASEBA School-age Forms and Profiles.

[bb0010] Adamson S.J., Kay-Lambkin F.J., Baker A.L., Lewin T.J., Thornton L., Kelly B.J., Sellman J.D. (2010). An improved brief measure of cannabis misuse: the Cannabis Use Disorders Identification Test-Revised (CUDIT-R). Drug Alcohol Depend..

[bb0015] Ahmadi A., Pearlson G.D., Meda S.A., Dager A., Potenza M.N., Rosen R., Austad C.S., Raskin S.A., Fallahi C.R., Tennen H., Wood R.M., Stevens M.C. (2013). Influence of alcohol use on neural response to go/no-go task in college drinkers. Neuropsychopharmacology.

[bb0020] Amunts K., Kedo O., Kindler M., Pieperhoff P., Mohlberg H., Shah N.J., Habel U., Schneider F., Zilles K. (2005). Cytoarchitectonic mapping of the human amygdala, hippocampal region and entorhinal cortex: intersubject variability and probability maps. Anat. Embryol..

[bb0025] Angold A., Costello E.J., Messer S.C., Pickles A., Winder F., Silver D. (1995). The development of a short questionnaire for use in epidemiological studies of depression in children and adolescents. Int. J. Methods Psychiatr. Res..

[bb0030] Babor T.F., Hofmann M., DelBoca F.K., Hesselbrock V., Meyer R.E., Dolinsky Z.S., Rounsaville B. (1992). Types of alcoholics, I. Evidence for an empirically derived typology based on indicators of vulnerability and severity. Arch. Gen. Psychiatry.

[bb0035] Birmaher B., Khetarpal S., Brent D., Cully M., Balach L., Kaufman J., Neer S.M. (1997). The Screen for Child Anxiety Related Emotional Disorders (SCARED): scale construction and psychometric characteristics. J. Am. Acad. Child Adolesc. Psychiatry.

[bb0040] Blair K.S., Smith B.W., Mitchell D.G.V., Morton J., Vythilingam M., Pessoa L., Fridberg D., Zametkin A., Nelson E.E., Drevets W.C., Pine D.S., Martin A., Blair R.J.R. (2007). Modulation of emotion by cognition and cognition by emotion. NeuroImage.

[bb0045] Blair K.S., Geraci M., Smith B.W., Hollon N., DeVido J., Otero M., Blair J.R., Pine D.S. (2012). Reduced dorsal anterior cingulate cortical activity during emotional regulation and top-down attentional control in generalized social phobia, generalized anxiety disorder, and comorbid generalized social phobia/generalized anxiety disorder. Biol. Psychiatry.

[bb0050] Blair K.S., Vythilingam M., Crowe S.L., Mccaffrey D.E., Ng P., Wu C.C., Mondillo K., Pine D.S., Charney D.S., Blair R.J.R., Services H. (2013). Cognitive control of attention is differentially affected in trauma exposed individuals with and without post-traumatic stress disorder. Psychol. Med..

[bb0055] Blom G. (1958). Statistical Estimates and Transformed Beta-variables.

[bb0060] Caldwell L.C., Schweinsburg A.D., Nagel B.J., Valerie C., Brown S.A., Tapert S.F. (2005). Gender and adolescent alcohol use disorders on BOLD response to spatial working memory. Alcohol Alcohol..

[bb0065] Claus E.D., Feldstein Ewing S.W., Filbey F.M., Hutchison K.E. (2013). Behavioral control in alcohol use disorders: relationships with severity. J. Stud. Alcohol Drugs.

[bb0070] Costafreda S.G., Brammer M.J., David A.S., Fu C.H.Y. (2008). Predictors of amygdala activation during the processing of emotional stimuli: a meta-analysis of 385 PET and fMRI studies. Brain Res. Rev..

[bb0075] Cox R.W. (1996). AFNI: software for analysis and visualization of functional magnetic resonance neuroimages. Comput. Biomed. Res..

[bb0080] Cox R.W., Chen G., Glen D.R., Reynolds R.C., Taylor P.A. (2017). FMRI clustering and false positive rates. Proc. Natl. Acad. Sci. U. S. A..

[bb0085] Criaud M., Boulinguez P. (2013). Have we been asking the right questions when assessing response inhibition in go/no-go tasks with fMRI? A meta-analysis and critical review. Neurosci. Biobehav. Rev..

[bb0090] Czapla M., Simon J.J., Richter B., Kluge M., Friederich H.C., Herpertz S., Mann K., Herpertz S.C., Loeber S. (2016). The impact of cognitive impairment and impulsivity on relapse of alcohol-dependent patients: implications for psychotherapeutic treatment. Addict. Biol..

[bb0095] Desimone R., Duncan J. (1995). Neural mechanisms of selective visual attention. Annu. Rev. Neurosci..

[bb0100] DeWitt S.J., Ketcherside A., McQueeny T.M., Dunlop J.P., Filbey F.M. (2015). The hyper-sentient addict: an exteroception model of addiction. Am. J. Drug Alcohol Abuse.

[bb0105] Erthal F.S., De Oliveira L., Mocaiber I., Pereira M.G., Machado-Pinheiro W., Volchan E., Pessoa L. (2005). Load-dependent modulation of affective picture processing. Cogn. Affect. Behav. Neurosci..

[bb0110] Fairlie A.M., Sindelar H.A., Eaton C.A., Spirito A. (2006). Utility of the AUDIT for screening adolescents for problematic alcohol use in the emergency department. Int. J. Adolesc. Med. Health.

[bb0115] Feldstein Ewing S.W., Sakhardande A., Blakemore S.J. (2014). The effect of alcohol consumption on the adolescent brain: a systematic review of MRI and fMRI studies of alcohol-using youth. NeuroImage Clin..

[bb0120] Filbey F., Yezhuvath U. (2013). Functional connectivity in inhibitory control networks and severity of cannabis use disorder. Am. J. Drug Alcohol Abuse.

[bb0125] Filbey F.M., McQueeny T., DeWitt S.J., Mishra V. (2015). Preliminary findings demonstrating latent effects of early adolescent marijuana use onset on cortical architecture. Dev. Cogn. Neurosci..

[bb0130] Gass J.T., Bailey W.G., Mcgonigal J.T., Trantham-Davidson H., Lopez M.F., Randall P.K., Yaxley R., Floresco S.B., Chandler L.J. (2014). Adolescent alcohol exposure reduces behavioral flexibility, promotes disinhibition, and increases resistance to extinction of ethanol self-administration in adulthood. Neuropsychopharmacology.

[bb0135] Gilman J.M., Hommer D.W. (2008). Modulation of brain response to emotional images by alcohol cues in alcohol-dependent patients. Addict. Biol..

[bb0140] Goddings A.L., Mills K.L., Clasen L.S., Giedd J.N., Viner R.M., Blakemore S.J. (2014). The influence of puberty on subcortical brain development. NeuroImage.

[bb0145] Gruber S.A., Yurgelun-Todd D.A. (2005). Neuroimaging of marijuana smokers during inhibitory processing: a pilot investigation. Cogn. Brain Res..

[bb0150] Gruber S.A., Rogowska J., Yurgelun-Todd D.A. (2010). Altered affective response in marijuana smokers: an FMRI study. Drug Alcohol Depend..

[bb0155] Gruber S.A., Dahlgren M.K., Sagar K.A., Gonenc A., Killgore W.D.S. (2013). Age of onset of marijuana use impacts inhibitory processing. Neurosci. Lett..

[bb9510] Hwang S., White S.F., Nolan Z.T., Sinclair S., Blair R.J.R. (2014). Neurodevelopmental changes in the responsiveness of systems involved in top down attention and emotional responding. Neuropsychologia.

[bb0160] Hwang S., White S.F., Nolan Z.T., Craig Williams W., Sinclair S., Blair R.J.R. (2015). Executive attention control and emotional responding in attention-deficit/hyperactivity disorder - a functional MRI study. NeuroImage Clin..

[bb0165] Hwang S., Nolan Z.T., White S.F., Williams W.C., Sinclair S., Blair R.J.R. (2016). Dual neuro-circuitry dysfunctions in disruptive behavior disorders: emotional responding and response inhibition. Psychol. Med..

[bb0170] (2012). IBM SPSS Statistics for MacOSX.

[bb0175] Irimia C., Wiskerke J., Natividad L.A., Polis I.Y., De Vries T.J., Pattij T., Parsons L.H. (2015). Increased impulsivity in rats as a result of repeated cycles of alcohol intoxication and abstinence. Addict. Biol..

[bb0180] Ketcherside A., Baine J., Filbey F. (2016). Sex effects of marijuana on brain structure and function. Curr. Addict. Rep..

[bb0185] Koob G.F., Volkow N.D. (2016). Neurobiology of addiction: a neurocircuitry analysis. Lancet Psychiatry.

[bb0190] Kowalski C.J., Schneiderman E.D., Willis S.M. (1994). ANCOVA for nonparallel slopes: the Johnson-Neyman technique. Int. J. Biomed. Comput..

[bb0195] Lang P., Greenwald M., Bradley M. (1988). The international affective picture system standardization procedure and initial group results for affective judgments: technical report 1B. J. Adolesc. Health.

[bb0200] Lee I.A., Preacher K.J. (2013). Calculation for the Test of the Difference Between Two Dependent Correlations With one Variable in Common.

[bb0205] Li C.R., Luo X., Yan P., Bergquist K., Sinha R. (2009). Altered impulse control in alcohol dependence: neural measures of stop signal performance. Alcohol. Clin. Exp. Res..

[bb0210] Luijten M., Machielsen M.W.J., Veltman D.J., Hester R., de Haan L., Franken I.H.A. (2014). Systematic review of ERP and fMRI studies investigating inhibitory control and error processing in people with substance dependence and behavioural addictions. J. Psychiatry Neurosci..

[bb0215] Mason W.A., Chmelka M.B., Howard B.K., Thompson R.W. (2013). Comorbid alcohol and cannabis use disorders among high-risk youth at intake into residential care. J. Adolesc. Health.

[bb0220] Miech R.A., Johnston L.D., O'Malley P.M., Bachman J.G., Schulenberg J.E. (2016). Monitoring the Future National Survey Results on Drug Use, 1975–2015: Volume I, Secondary School Students.

[bb0225] Mitterschiffthaler M.T., Williams S.C.R., Walsh N.D., Cleare A.J., Donaldson C., Scott J., Fu C.H.Y. (2008). Neural basis of the emotional Stroop interference effect in major depression. Psychol. Med..

[bb0230] Moss H.B., Chen C.M., Yi H. ye (2014). Early adolescent patterns of alcohol, cigarettes, and marijuana polysubstance use and young adult substance use outcomes in a nationally representative sample. Drug Alcohol Depend..

[bb0235] Nikolova Y., Knodt A., Radtke S., Hariri A. (2016). Divergent responses of the amygdala and ventral striatum predict stress-related problem drinking in young adults: possible differential markers of affective and impulsive pathways of risk for alcohol use disorder. Mol. Psychiatry.

[bb0240] Norman A.L., Pulido C., Squeglia L.M., Spadoni A.D., Paulus M.P., Tapert S.F. (2011). Neural activation during inhibition predicts initiation of substance use in adolescence. Drug Alcohol Depend..

[bb0245] Ochsner K.N., Gross J.J. (2005). The cognitive control of emotion. Trends Cogn. Sci..

[bb0250] O'Daly O.G., Trick L., Scaife J., Marshall J., Ball D., Phillips M.L., Williams S.S.C., Stephens D.N., Duka T. (2012). Withdrawal-associated increases and decreases in functional neural connectivity associated with altered emotional regulation in alcoholism. Neuropsychopharmacology.

[bb0255] Peters S., Jolles D.J., Van Duijvenvoorde A.C.K., Crone E.A., Peper J.S. (2015). The link between testosterone and amygdala-orbitofrontal cortex connectivity in adolescent alcohol use. Psychoneuroendocrinology.

[bb0260] Preacher K.J., Hayes A.F. (2004). SPSS and SAS procedures for estimating indirect effects in simple mediation models. Behav. Res. Methods Instrum. Comput..

[bb0265] Saunders J.B., Aasland O.G., Babor T.F., de la Fuente J.R., Grant M. (1993). Development of the Alcohol Use Disorders Identification Test (AUDIT): WHO collaborative project on early detection of persons with harmful alcohol consumption—II. Addiction.

[bb0270] Silveri M.M., Dager A.D., Cohen-Gilbert J.E., Sneider J.T. (2016). Neurobiological signatures associated with alcohol and drug use in the human adolescent brain. Neurosci. Biobehav. Rev..

[bb0275] Spear L.P. (2016). Consequences of adolescent use of alcohol and other drugs: studies using rodent models. Neurosci. Biobehav. Rev..

[bb0280] Spechler P.A., Orr C.A., Chaarani B., Kan K.J., Mackey S., Morton A., Snowe M.P., Hudson K.E., Althoff R.R., Higgins S.T., Cattrell A., Flor H., Nees F., Banaschewski T., Bokde A.L.W., Whelan R., Büchel C., Bromberg U., Conrod P., Frouin V., Papadopoulos D., Gallinat J., Heinz A., Walter H., Ittermann B., Gowland P., Paus T., Poustka L., Martinot J.L., Artiges E., Smolka M.N., IMAGEN Consortium, Garavan H. (2015). Cannabis use in early adolescence: evidence of amygdala hypersensitivity to signals of threat. Dev. Cogn. Neurosci..

[bb0285] Squeglia L.M., Tapert S.F., Sullivan E.V., Jacobus J., Meloy M.J., Rohlfing T., Pfefferbaum A. (2015). Brain development in heavy-drinking adolescents. Am. J. Psychiatry.

[bb0290] Squire R.F., Noudoost B., Schafer R.J., Moore T. (2013). Prefrontal contributions to visual selective attention. Annu. Rev. Neurosci..

[bb0295] Steiger J.H. (1980). Tests for comparing elements of a correlation matrix. Psychol. Bull..

[bb0300] Talairach J., Tournoux P. (1988). Co-planar Stereotaxic Atlas of the Human Brain: 3-D Proportional System: An Approach to Cerebral Imaging.

[bb0305] Tapert S.F., Schweinsburg A.D., Drummond S.P. a, Paulus M.P., Brown S. a, Yang T.T., Frank L.R. (2008). Functional MRI of inhibitory processing in abstinent adolescent marijuana users. Psychopharmacology.

[bb0310] Thayer R.E., Feldstein Ewing S.W., Dodd A.B., Hansen N.S., Mayer A.R., Ling J.M., Bryan A.D. (2015). Functional activation during the Stroop is associated with recent alcohol but not marijuana use among high-risk youth. Psychiatry Res. Neuroimaging.

[bb0315] Volkow N.D., Koob G.F., McLellan A.T. (2016). Neurobiologic advances from the brain disease model of addiction. N. Engl. J. Med..

[bb0320] Wagner G., Sinsel E., Sobanski T., Köhler S., Marinou V., Mentzel H.J., Sauer H., Schlösser R.G.M. (2006). Cortical inefficiency in patients with unipolar depression: an event-related fMRI study with the Stroop task. Biol. Psychiatry.

[bb0325] Wetherill R.R., Squeglia L.M., Yang T.T., Tapert S.F. (2014). A longitudinal examination of adolescent response inhibition: neural differences before and after the initiation of heavy drinking. Psychopharmacology.

[bb0330] White S.F., Costanzo M.E., Blair J.R., Roy M.J. (2014). PTSD symptom severity is associated with increased recruitment of top-down attentional control in a trauma-exposed sample. NeuroImage Clin..

[bb0335] Winters K.C., Lee C.Y.S. (2008). Likelihood of developing an alcohol and cannabis use disorder during youth: association with recent use and age. Drug Alcohol Depend..

[bb0340] Zakiniaeiz Y., Scheinost D., Seo D., Sinha R., Constable R.T. (2017). Cingulate cortex functional connectivity predicts future relapse in alcohol dependent individuals. NeuroImage Clin..

