# Supplementary material for: Adolescents show differential dysfunctions related to Alcohol and Cannabis Use Disorder severity in emotion and executive attention neuro-circuitries
Source: Neuroimage Clin. 2018 Jun 5;19:782–92. doi: 10.1016/j.nicl.2018.06.005 (PMC6031867; doi:10.1016/j.nicl.2018.06.005)
Supplement: Supplementary file 1 — Supplementary material [file mmc1.docx]

Supplemental Results

The AFNI ClustSim program was used to establish a *p*=.05 corrected threshold (19 voxel clusters at initial threshold of *p*=.001) for a whole-brain analysis. All reported regions in the whole-brain analysis exceed this threshold.

*Main Effect of AUDIT Score*

No brain regions survived correction for multiple comparisons.

*Main Effect of CUDIT Score*

No brain regions survived correction for multiple comparisons.

*Main Effect of Emotion*

There was a main effect of emotion in dorsolateral prefrontal cortex (dlPFC), inferior frontal gyrus (iFG), precentral gyrus, inferior parietal lobule (iPL), precuneus, parahippocampal gyrus (PHG), superior temporal gyrus (STG), middle temporal gyrus (MTG), fusiform gyrus, amygdala, and cerebellum. For further details, including all contrasts significant at *p*<0.05, see Table S1.

*Main Effect of Task Condition*

There was a main effect of task condition in cerebellum, visual, parietal, frontal, insular, and motor cortices, medial prefrontal cortex (mPFC), dlPFC, iFG, precentral gyrus, supplementary motor area (SMA), iPL, posterior cingulate cortex (PCC), angular gyrus, inferior temporal gyrus (iTG), MTG, PHG, and amygdala. For further details, including all contrasts significant at *p*<0.05, see Table S1 and Figure S1.

*AUDIT by CUDIT Score Interaction Effect*

No brain regions survived correction for multiple comparisons.

*AUDIT by Emotion Interaction Effect*

No brain regions survived correction for multiple comparisons.

*CUDIT by Emotion Interaction Effect*

No brain regions survived correction for multiple comparisons.

*AUDIT by CUDIT by Emotion Interaction Effect*

No brain regions survived correction for multiple comparisons.

*AUDIT by CUDIT by Task Condition Interaction Effect*

No brain regions survived correction for multiple comparisons.

*Emotion by Task Condition Interaction Effect*

There were emotion by task condition interaction effects in lentiform nucleus/putamen, anterior cingulate/ventromedial prefrontal cortex (ACC/vmPFC), dorsolateral prefrontal cortex, and cingulate gyrus. In the lentiform nucleus/putamen cluster, there was increased activity for task relative to view trials for negative and neutral stimuli [all *t*'s>3.13, all *p*'s<0.01]. Additionally, there was increased activity for congruent relative to incongruent trials for negative stimuli [*t*(81)=3.24, *p*<0.05]. There was increased activity for incongruent relative to congruent and view trials for positive stimuli [*t*(81)=2.10 and 3.09, respectively; both *p*'s<0.05]. In the ACC/vmPFC cluster, there was decreased activity for incongruent relative to view and congruent trials for negative stimuli [*t*(81)=-5.98 and -3.83, respectively; both *p*'s<0.001]. For neutral and positive stimuli, there was decreased activity for task relative to view trials [*t*'s<-2.34; all *p*'s<0.05]. For positive stimuli, there was increased activity for incongruent relative to congruent trials [*t*=2.12, *p*<.05]. In the dlPFC cluster, there was increased activity for task relative to view trials for negative and neutral stimuli [all *t*'s>2.18, all *p*'s<0.05]. Furthermore, for positive stimuli, there was increased BOLD response for incongruent relative to view trials [*t*(81)=2.34, *p*<0.05]. In the cingulate gyrus cluster, there was increased activity for task relative to view trials for all stimuli [all *t*'s>4.58, all *p*'s<0.001]. Additionally, there was increased activity for incongruent relative to congruent trials for positive stimuli [*t*(81)=2.16, *p<*0.05]. For more details, see Table S1.

*AUDIT by Emotion by Task condition Interaction Effect*

No brain regions survived correction for multiple comparisons.

*CUDIT by Emotion by Task Condition Interaction Effect*

No brain regions survived correction for multiple comparisons.

*Supplemental Results: Age Covariate (Table S2)*

Since age was significantly related to AUDIT score within this sample, the analysis was repeated with age as a covariate. The AFNI ClustSim program was used to establish a *p*=.05 corrected threshold (5 voxel clusters at initial threshold of *p*=.02) for the amygdala. All reported regions in the amygdala ROI exceed this threshold. The AFNI ClustSim program was used to establish a *p*=.05 corrected threshold (19 voxel clusters at initial threshold of *p*=.001) for a whole-brain analysis. All reported regions in the whole-brain analysis exceed this threshold.

*Amygdala ROI:*

There was a significant AUDIT by emotion interaction effect in the right amygdala (k=5 voxels) and a significant AUDIT by CUDIT by emotion by task condition interaction effect in the left amygdala (k=10 voxels). Both of these clusters were overlapped with clusters that were significant for the amygdala ROI analysis in the main analysis.

*AUDIT by Task Condition Interaction Effect*

There were significant AUDIT by task condition interaction effects in the dlPFC, superior frontal gyrus (SFG), anterior cingulate cortex/dorsomedial prefrontal cortex (ACC/dmPFC), PCC, precuneus, iPL, MTG, and STG. Of these clusters, the dlPFC, ACC/dmPFC, PCC, precuneus, iPL, and MTG clusters overlapped with significant clusters for this contrast in the main analysis.

*CUDIT by Task Condition Interaction Effects*

There were significant CUDIT by task condition interaction effects in the PCC, precuneus, MTG, STG, and culmen. All clusters overlapped with significant clusters for this contrast in the main analysis.

*AUDIT by CUDIT by Emotion by Task Condition*

There was a significant cluster in inferior frontal gyrus (iFG). The iFG cluster overlapped with the significant iFG cluster for this contrast in the main analysis.

*Supplemental Results: Multivariate Outliers Removed (Table S3)*

Mahalanobis distances (MD) were calculated for each participant in order to calculate how many standard deviations each participant is away from the mean of our sample distribution within our variable space. We calculated the MD for each participant on AUDIT scores and CUDIT scores. We then calculated a *p*-value for each participant under a chi-square distribution to determine whether their MD value represents a multivariate outlier. Four participants were identified as multivariate outliers using a significance threshold of *p*<.001; therefore, the analysis was repeated without these participants in the sample. The AFNI ClustSim program was used to establish a *p*=.05 corrected threshold (5 voxel clusters at initial threshold of *p*=.02) for the amygdala. All reported regions in the amygdala ROI exceed this threshold. The AFNI ClustSim program was used to establish a *p*=.05 corrected threshold (19 voxel clusters at initial threshold of *p*=.001) for a whole-brain analysis. All reported regions in the whole-brain analysis exceed this threshold.

*Amygdala ROI:*

There was a significant AUDIT by emotion interaction effect in the right amygdala (*k*=6 voxels). This cluster overlapped with the AUDIT-by-emotion cluster that was in the main analysis.

*AUDIT by Task Condition Interaction Effect*

There were significant AUDIT by task condition interaction effects in dlPFC, MFG, ACC, dmPFC, PCC, precuneus, iPL, postcentral gyrus, supramarginal gyrus, and cerebellum. All clusters (except postcentral gyrus) overlapped with significant clusters for this contrast in the main analysis.

*CUDIT by Task Condition Interaction Effect*

There were significant CUDIT by task condition interaction effects in PCC, precuneus, iPL, precentral gyrus, and cerebellum. All clusters (except precentral gyrus) overlapped with significant clusters for this contrast in the main analysis.

*CUDIT by Emotion by Task Condition Interaction Effect*

There was a significant CUDIT by emotion by task condition interaction effect in the right superior parietal lobule (SPL). This cluster did not overlap with any significant clusters for this contrast in the main analysis.

*AUDIT by CUDIT by Emotion by Task Interaction Effect*

There was a significant AUDIT by CUDIT by emotion by task condition interaction effect in the cuneus. This cluster did not overlap with any significant clusters for this contrast in the main analysis.

*CUDIT by Emotion Interaction Effect*

There was a CUDIT by emotion interaction effect in the lentiform nucleus/putamen. This cluster did not overlap with any significant clusters for this contrast in the main analysis.

*AUDIT by CUDIT by Emotion Interaction Effect*

There was an AUDIT-by-CUDIT-by-Emotion interaction effect in the dlPFC, middle frontal gyrus (MFG), ventromedial prefrontal cortex (vmPFC), STG, MTG, and fusiform gyrus. These clusters did not overlap with any significant clusters for this contrast in the main analysis.

*Supplemental Results: Gender (Table S4)*

Since alcohol and cannabis may differentially affect males versus females, the main analysis was repeated with gender added as a between-subjects variable. The AFNI ClustSim program was used to establish a *p*=.05 corrected threshold (5 voxel clusters at initial threshold of *p*=.02) for the amygdala. All reported regions in the amygdala ROI exceed this threshold. The AFNI ClustSim program was used to establish a *p*=.05 corrected threshold (19 voxel clusters at initial threshold of *p*=.001) for a whole-brain analysis. All reported regions in the whole-brain analysis exceed this threshold.

*Amygdala ROI:*

There was a significant AUDIT by emotion interaction effect in the right amygdala (*k*=6 voxels). This cluster overlapped with the AUDIT-by-emotion cluster that was in the main analysis. There was a significant AUDIT-by-CUDIT-by-emotion-by-task condition interaction in the left amygdala (*k*=9 voxels). This cluster overlapped with the AUDIT-by-CUDIT-by-emotion-by-task condition interaction in the main analysis.

*AUDIT by Task Condition Interaction Effect*

There were significant AUDIT-by-task condition interaction effects in the dlPFC, iFG, ACC, dmPFC, SMA, PCC, precuneus, iPL, postcentral gyrus, supramarginal gyrus, and cerebellum. All but one cluster (SMA) overlapped with clusters for this contrast in the main analysis.

*CUDIT by Task Condition Interaction Effect*

There were significant CUDIT-by-task condition interaction effects in PCC, iPL, MTG, and culmen. All clusters overlapped with clusters for this contrast in the main analysis.

*AUDIT by CUDIT by Emotion by Task Interaction Effect*

There was a significant AUDIT-by-CUDIT-by-emotion-by-task condition interaction effect in the iFG. This cluster overlapped with the iFG cluster for this contrast in the main analysis.

*CUDIT by Emotion Interaction Effect*

There were CUDIT-by-emotion interaction effects in the putamen and cerebellum. These clusters did not overlap with any clusters in the main analysis.

*Supplemental Results: Non-Smokers (Table S5)*

To rule out the possibility that our results were related to smoking, the analysis was repeated with participants who endorsed current regular smoking excluded from the sample (N=10, resulting in a non-smoking sample size of N=72). The AFNI ClustSim program was used to establish a *p*=.05 corrected threshold (5 voxel clusters at initial threshold of *p*=.02) for the amygdala. All reported regions in the amygdala ROI exceed this threshold. The AFNI ClustSim program was used to establish a *p*=.05 corrected threshold (19 voxel clusters at initial threshold of *p*=.001) for a whole-brain analysis. All reported regions in the whole-brain analysis exceed this threshold.

*Amygdala ROI:*

There was a significant AUDIT-by-CUDIT-by-emotion-by-task interaction in the left amygdala (*k*=23 voxels). This cluster overlapped with the cluster found for this contrast in the main analysis.

*AUDIT by Task Condition Interaction Effect*

There were significant AUDIT-by-task condition interaction effects in the dlPFC, MFG, ACC, PCC, precuneus, MTG, PHG, and culmen. All clusters overlapped with clusters found for this contrast in the main analysis.

*CUDIT by Task Condition Interaction Effect*

There were significant CUDIT-by-task condition interaction effects in PCC, parahippocampal gyrus, and culmen. The PCC and culmen clusters overlapped with clusters fround for this contrast in the main analysis.

*AUDIT by CUDIT by Emotion by Task Interaction Effect*

There was a significant AUDIT-by-CUDIT-by-emotion-by-task condition interaction effect in dlPFC, MFG, iFG, precentral gyrus, STG, PHG, and cerebellum. No clusters overlapped with the cluster for this contrast in the main analysis.

*Supplemental Results: Substance Users Only (Table S6)*

In order to rule out the possibility that over-representation of 0 for AUDIT and CUDIT scores biased our results, we re-ran the ANCOVA analysis in individuals reported alcohol and/or cannabis use only. Since there were only 49 participants at this sample, we used an initial threshold of *p*=.05 for the amygdala ROI analysis and *p*=.002 for the whole-brain analysis. The AFNI ClustSim program was used to establish a *p*=.05 corrected threshold (9 voxel clusters at initial threshold of *p*=.05) for the amygdala. All reported regions in the amygdala ROI exceed this threshold. The AFNI ClustSim program was used to establish a *p*=.05 corrected threshold (27 voxel clusters at initial threshold of *p*=.002) for a whole-brain analysis. All reported regions in the whole-brain analysis exceed this threshold.

*Amygdala ROI:*

There was a significant AUDIT-by-emotion interaction in the right amygdala (*k*=15 voxels). There was a significant AUDIT-by-CUDIT-by-emotion-by-task condition interaction in the left amygdala (*k*=17 voxels). Both clusters overlapped with clusters found for these contrasts in the main analysis.

*AUDIT by Task Condition Interaction Effect*

There were significant AUDIT-by-task condition interaction effects in dlPFC, ACC, anterior insula cortex (aIC), PCC, precuneus, iPL, STG, occipital cortex, and cerebellum. The clusters within dlPFC, ACC, PCC, precuneus, iPL, dlPFC, ACC, PCC, precuneus, iPL, and cerebellum overlapped with clusters found for this contrast in the main analysis.

*CUDIT by Task Condition Interaction Effect*

There was a significant CUDIT-by-task condition interaction effect in precuneus. This cluster overlapped with a cluster found for this contrast in the main analysis.

*Supplemental Results: RT Included in the Model (Table S7)*

To see whether reaction time (RT) explained some of the variance in ACC and dmPFC responses during the aST, the main analysis was repeated with average RT as a covariate. The AFNI ClustSim program was used to establish a *p*=.05 corrected threshold (5 voxel clusters at initial threshold of *p*=.02) for the amygdala. All reported regions in the amygdala ROI exceed this threshold. The AFNI ClustSim program was used to establish a *p*=.05 corrected threshold (19 voxel clusters at initial threshold of *p*=.001) for a whole-brain analysis. Unless otherwise noted, all reported regions in the whole-brain analysis exceed this threshold.

*Amygdala ROI:*

There was a significant AUDIT-by-emotion interaction in the right amygdala (*k*=5 voxels). There was a significant AUDIT-by-CUDIT-by-emotion-by-task condition interaction in the left amygdala (*k*=9 voxels). Both clusters overlapped with clusters found for these contrasts in the main analysis.

*AUDIT by Task Condition Interaction Effect*

There were significant AUDIT-by-task condition effects in dlPFC, MFG, iFG, ACC, dmPFC,PCC, precuneus, iPL, postcentral gyrus, MTG, supramarginal gyrus, PHG, and cerebellum. All clusters overlapped with clusters found for this contrast in the main analysis.

*CUDIT by Task Condition Interaction Effect*

There were significant CUDIT-by-task condition effects in PCC, precuneus, iPL, MTG, and culmen,. All clusters overlapped with clusters found for this contrast in the main analysis.

*RT by Task Condition Interaction Effect*

There were no regions that survived comparisons for multiple corrections in this contrast; however, there were two subthreshold clusters within aIC/iFG and dmPFC.

*AUDIT by CUDIT by Emotion by Task Interaction Effect*

There was a significant AUDIT-by-CUDIT-by-emotion-by-task condition interaction effect in the iFG. This cluster overlapped with the iFG cluster for this contrast in the main analysis.

| Table S1. Brain Regions demonstrating significant Main Effects of Emotion, Main Effects of Task Condition, Emotion by Task Condition Interaction Effects | | | | | | | | |  |
| --- | --- | --- | --- | --- | --- | --- | --- | --- | --- |
| Coordinates of Peak Activation^b^ | | | | | | | | |  |
| Region^a^ | Hemisphere | BA | x | y | z | *F* | Partial η^2^ | Voxels |  |
| Main Effect of Emotion | | | | | | | | |  |
| Significant Contrasts: Negative > Positive > Neutral | | | | | | | | |  |
| Fusiform Gyrus | R | 37 | 41 | -43 | -16 | 70.06 | 0.473 | 1092 |  |
| Fusiform Gyrus | L | 20 | -37 | -40 | -16 | 64.64 | 0.453 | 993 |  |
| Uncus | R | 21 | 35 | -4 | -28 | 16.72 | 0.177 | 22 |  |
| Amygdala | R | - | 20 | -1 | -16 | 16.45 | 0.174 | 21 |  |
| Significant Contrasts: Negative > Neutral = Positive | | | | | | | | |  |
| iFG | L | 47 | -31 | 29 | -10 | 16.45 | 0.174 | 22 |  |
| iFG | R | 46 | 50 | 29 | 17 | 15.49 | 0.166 | 19 |  |
| Significant Contrasts: Positive = Neutral > Negative | | | | | | | | |  |
| dlPFC | R | 10 | 35 | 56 | 5 | 12.24 | 0.136 | 24 |  |
| iPL | R | 40 | 56 | -34 | 44 | 17.14 | 0.180 | 151 |  |
| iPL | L | 40 | -55 | -31 | 44 | 14.23 | 0.154 | 30 |  |
| Superior Temporal Gyrus | L | 41 | -58 | -25 | 11 | 13.43 | 0.147 | 22 |  |
| Significant Contrasts: Neutral > Positive = Negative | | | | | | | | |  |
| Precuneus | L | 7 | -13 | -61 | 50 | 10.81 | 0.122 | 21 |  |
| Parahippocampal Gyrus | R | 19 | 29 | -43 | -4 | 23.19 | 0.229 | 43 |  |
| Significant Contrasts: Neutral > Positive > Negative | | | | | | | | |  |
| aIC/Precentral Gyrus/iFG | R | 13 | 47 | 11 | 2 | 16.69 | 0.176 | 42 |  |
| Parahippocampal Gyrus | L | 36 | -25 | -43 | -7 | 27.55 | 0.261 | 48 |  |
| Superior Temporal Gyrus | R | 22 | 62 | -13 | 5 | 16.52 | 0.175 | 55 |  |
| Superior Temporal Gyrus | L | 22 | -55 | -7 | 8 | 14.80 | 0.159 | 49 |  |
| Main Effect of Task Condition | | | | | | | | |  |
| Significant Contrasts: Incongruent = Congruent > View | | | | | | | | |  |
| Cerebellum/Visual/Parietal/Motor/Insular/Frontal Cortex | R/L |  | -10 | -22 | 5 | 139.88 | 0.642 | 14601 |  |
| dlPFC | R | 10 | 35 | 38 | 20 | 29.96 | 0.250 | 260 |  |
| Cingulate Gyrus | R | 23 | 5 | -28 | 26 | 35.63 | 0.314 | 140 |  |
| Significant Contrasts: View > Incongruent = Congruent | | | | | | | | |  |
| Medial Prefrontal Cortex | R/L | 24 | -4 | 23 | -4 | 26.62 | 0.254 | 904 |  |
| iFG | R | 46 | 53 | 32 | 11 | 26.30 | 0.252 | 97 |  |
| iFG | R | 47 | 26 | 29 | -4 | 13.08 | 0.144 | 19 |  |
| Supplementary Motor Area | R/L | 6 | 5 | -25 | 59 | 19.13 | 0.197 | 115 |  |
| Precentral Gyrus | R | 4 | 29 | -25 | 50 | 22.15 | 0.221 | 83 |  |
| Precentral Gyrus | L | 6 | -43 | -13 | 35 | 15.77 | 0.168 | 26 |  |
| PCC | L | 29 | -7 | -49 | 11 | 12.38 | 0.137 | 22 |  |
| PCC | L | 31 | -10 | -40 | 35 | 15.81 | 0.169 | 44 |  |
| iPL | R | 39 | 50 | -61 | 41 | 16.20 | 0.172 | 86 |  |
| Angular Gyrus | L | 39 | -49 | -64 | 32 | 25.09 | 0.243 | 233 |  |
| Middle Temporal Gyrus | L | 21 | -61 | -37 | 2 | 14.41 | 0.156 | 35 |  |
| Middle Temporal Gyrus | R | 21 | 59 | -37 | -1 | 15.77 | 0.168 | 34 |  |
| Middle Temporal Gyrus | R | 21 | 53 | -1 | -13 | 15.27 | 0.164 | 34 |  |
| Inferior Temporal Gyrus | L | 21 | -55 | -13 | -16 | 19.12 | 0.197 | 117 |  |
| Significant Contrasts: View > Congruent > Incongruent | | | | | | | | |  |
| iFG | L | 45 | -49 | 29 | 8 | 44.41 | 0.363 | 262 |  |
| Parahippocampal Gyrus/Amygdala | L | 36 | -31 | -28 | -13 | 28.64 | 0.269 | 127 |  |
| Parahippocampal Gyrus/Amygdala | R | - | 20 | -10 | -16 | 18.36 | 0.191 | 37 |  |
| Emotion by Task Condition | | | | | | | | |  |
| Lentiform Nucleus/Putamen | R | - | 20 | 11 | -7 | 9.41 | 0.108 | 22 |  |
| ACC/vmPFC | R/L | 24 | 5 | 32 | -1 | 6.94 | 0.082 | 20 |  |
| dlPFC | R | 9 | 26 | 32 | 32 | 6.55 | 0.078 | 23 |  |
| Cingulate Gyrus | R/L | 24 | -1 | -1 | 41 | 6.98 | 0.082 | 20 |  |

Note: ^a^ According to the Talairach Daemon Atlas (<http://www.nitrc.org/projects/tal-daemon/>), ^b^ Based on
the Tournoux & Talairach standard brain template, BA= Brodmann’s Area

| Table S2. Brain Regions demonstrating significant Interaction Effects Covarying for Age | | | | | | | | |  |
| --- | --- | --- | --- | --- | --- | --- | --- | --- | --- |
| Coordinates of Peak Activation^b^ | | | | | | | | |  |
| Region^a^ | Hemisphere | BA | x | y | z | *F* | Partial η^2^ | Voxels |  |
| AUDIT x Task Condition | | | | | | | | |  |
| dlPFC* | R | 8 | 29 | 35 | 44 | 18.93 | 0.197 | 130 |  |
| dlPFC | R | 9 | 32 | 44 | 29 | 13.88 | 0.153 | 49 |  |
| dlPFC* | L | 10 | -34 | 47 | 20 | 13.76 | 0.152 | 33 |  |
| dlPFC | L | 8 | -19 | 38 | 47 | 14.49 | 0.158 | 29 |  |
| MFG* | R | 6 | 20 | 20 | 56 | 14.30 | 0.157 | 31 |  |
| iFG/dlPFC* | R | 9 | 53 | 5 | 29 | 10.90 | 0.124 | 22 |  |
| ACC/dmPFC* | R/L | 6/32 | 2 | 11 | 44 | 15.17 | 0.165 | 60 |  |
| dmPFC | R/L | 6 | 8 | -1 | 62 | 12.74 | 0.142 | 22 |  |
| dmPFC | L | 6 | -4 | -7 | 56 | 14.77 | 0.161 | 19 |  |
| Precuneus/PCC* | R/L | 7/31 | 11 | -67 | 29 | 25.40 | 0.248 | 1759 |  |
| iPL* | R | 40 | 32 | -49 | 41 | 16.11 | 0.173 | 46 |  |
| iPL* | R | 13 | 50 | -43 | 23 | 14.03 | 0.154 | 29 |  |
| Postcentral Gyrus* | R | 41 | 53 | -19 | 14 | 12.68 | 0.141 | 33 |  |
| MTG* | R | 19 | 44 | -61 | 11 | 17.27 | 0.183 | 86 |  |
| Supramarginal Gyrus* | R | 40 | 53 | -37 | 35 | 16.16 | 0.173 | 24 |  |
| Parahippocampal Gyrus* | L | 27 | -25 | -34 | -1 | 18.28 | 0.192 | 29 |  |
| Culmen | L | - | -34 | -58 | -25 | 11.47 | 0.130 | 27 |  |
| CUDIT x Task Condition | | | | | | | | |  |
| PCC* | R/L | 31 | 11 | -52 | 26 | 14.67 | 0.160 | 80 |  |
| Precuneus* | R | 31 | 14 | -70 | 29 | 11.88 | 0.134 | 27 |  |
| Precuneus* | L | 31 | -16 | -67 | 26 | 12.51 | 0.140 | 26 |  |
| iPL* | R | 39 | 35 | -58 | 38 | 12.17 | 0.137 | 25 |  |
| MTG* | R | 39 | 50 | -67 | 26 | 11.22 | 0.127 | 26 |  |
| MTG* | R | 19 | 44 | -61 | 11 | 12.63 | 0.141 | 23 |  |
| Culmen* | L | - | -7 | -61 | -7 | 13.14 | 0.146 | 36 |  |
| Pyramis* | L | - | -31 | -67 | -34 | 12.84 | 0.143 | 21 |  |
|  |  |  |  |  |  |  |  |  |  |
| AUDIT x CUDIT x Emotion x Task Condition | | | | | | | | |  |
| iFG* | L | 9 | -40 | 5 | 29 | 7.17 | 0.085 | 40 |  |

Note: ^a^ According to the Talairach Daemon Atlas (<http://www.nitrc.org/projects/tal-daemon/>), ^b^ Based on
the Tournoux & Talairach standard brain template, * Denotes regions that overlap with and/or are proximal to
clusters found in the main analysis, BA= Brodmann’s Area

| Table S3. Brain Regions demonstrating significant Interaction Effects After Removing Multivariate Outliers | | | | | | | | |  |
| --- | --- | --- | --- | --- | --- | --- | --- | --- | --- |
| Coordinates of Peak Activation^b^ | | | | | | | | |  |
| Region^a^ | Hemisphere | BA | x | y | z | *F* | Partial η^2^ | Voxels |  |
| AUDIT x Task Condition | | | | | | | | |  |
| dlPFC* | R | 8 | 29 | 35 | 44 | 13.23 | 0.152 | 32 |  |
| dlPFC | L | 8/9 | -22 | 50 | 35 | 13.38 | 0.153 | 26 |  |
| MFG* | R | 6 | 20 | 20 | 56 | 12.69 | 0.146 | 23 |  |
| iFG/dlPFC* | R | 6 | 53 | 2 | 32 | 14.44 | 0.163 | 52 |  |
| ACC/dmPFC* | R/L | 6/32 | -1 | 11 | 44 | 17.37 | 0.190 | 150 |  |
| dmPFC | R | 6 | 8 | 2 | 53 | 13.79 | 0.157 | 25 |  |
| Precuneus/PCC* | R/L | 7/31 | 11 | -67 | 32 | 21.70 | 0.227 | 1332 |  |
| Precuneus* | R | 7 | 8 | -46 | 56 | 13.77 | 0.157 | 37 |  |
| Postcentral Gyrus | R | 2/3 | 41 | -31 | 59 | 17.87 | 0.195 | 146 |  |
| Postcentral Gyrus* | R | 41 | 50 | -19 | 14 | 11.53 | 0.135 | 19 |  |
| MTG* | R | 19 | 44 | -61 | 11 | 18.92 | 0.204 | 94 |  |
| STG* | R | 13 | 53 | -43 | 20 | 14.04 | 0.159 | 21 |  |
| CUDIT x Task Condition | | | | | | | | |  |
| Precentral Gyrus | R | 6 | 50 | -13 | 32 | 11.19 | 0.131 | 22 |  |
| Precuneus/PCC* | R/L | 31 | 11 | -55 | 26 | 22.42 | 0.233 | 323 |  |
| MTG* | R | 19 | 44 | -61 | 11 | 14.90 | 0.168 | 49 |  |
| Parahippocampal Gyrus | L | 35 | -19 | -31 | -7 | 20.59 | 0.218 | 28 |  |
| Cerebellum* | L | - | -31 | -64 | -34 | 18.32 | 0.198 | 125 |  |
| Culmen* | L | - | -1 | -58 | -4 | 17.54 | 0.192 | 106 |  |
| CUDIT x Emotion x Task Condition | | | | | | | | |  |
| Superior Parietal Lobule | R | 7 | 41 | -55 | 50 | 7.28 | 0.090 | 30 |  |
| AUDIT x CUDIT x Emotion x Task Condition | | | | | | | | |  |
| Cuneus | L | 17 | -4 | -76 | 14 | 6.36 | 0.079 | 25 |  |
| CUDIT x Emotion | | | | | | | | |  |
| Lentiform Nucleus/Putamen | R | - | 26 | 8 | 2 | 15.27 | 0.171 | 37 |  |
| AUDIT x CUDIT x Emotion | | | | | | | | |  |
| dlPFC | L | 10 | -25 | 62 | 8 | 20.49 | 0.217 | 38 |  |
| MFG | L | 6/8 | -28 | 11 | 41 | 17.20 | 0.189 | 76 |  |
| vmPFC | R/L | 11/12 | 0 | 38 | -12 | 18.71 | 0.202 | 43 |  |
| STG | L | 39 | -55 | -58 | 20 | 16.20 | 0.180 | 49 |  |
| MTG | L | 22 | -61 | -40 | 5 | 15.98 | 0.178 | 29 |  |
| Fusiform Gyrus | L | 20 | -37 | -37 | -16 | 19.38 | 0.208 | 19 |  |

Note: ^a^ According to the Talairach Daemon Atlas (<http://www.nitrc.org/projects/tal-daemon/>), ^b^ Based on
the Tournoux & Talairach standard brain template, * Denotes regions that overlap with and/or are proximal to
clusters found in the main analysis, BA= Brodmann’s Area

| Table S4. Brain Regions demonstrating significant Interaction Effects After Controlling for Gender | | | | | | | | |  |
| --- | --- | --- | --- | --- | --- | --- | --- | --- | --- |
| Coordinates of Peak Activation^b^ | | | | | | | | |  |
| Region^a^ | Hemisphere | BA | x | y | z | *F* | Partial η^2^ | Voxels |  |
| AUDIT x Task Condition | | | | | | | | |  |
| dlPFC* | R | 8 | 29 | 35 | 44 | 16.41 | 0.176 | 65 |  |
| iFG* | R | 44 | 53 | 5 | 23 | 10.15 | 0.116 | 19 |  |
| ACC/dmPFC* | R/L | 6/32 | 2 | 11 | 44 | 13.28 | 0.147 | 54 |  |
| SMA | R/L | 6 | -4 | -7 | 56 | 14.29 | 0.157 | 22 |  |
| Precuneus/PCC* | R/L | 7/31 | 14 | -70 | 29 | 21.82 | 0.221 | 1345 |  |
| iPL* | R | 40 | 35 | -49 | 41 | 13.68 | 0.151 | 22 |  |
| Postcentral Gyrus* | R | 41 | 53 | -19 | 14 | 11.88 | 0.134 | 27 |  |
| MTG* | R | 37 | 47 | -64 | 11 | 16.78 | 0.179 | 75 |  |
| Supramarginal Gyrus* | R | 40 | 50 | -37 | 35 | 15.47 | 0.167 | 21 |  |
| Cerebellum* | R/L | - | -1 | -61 | -28 | 14.53 | 0.159 | 37 |  |
| CUDIT x Task Condition | | | | | | | | |  |
| PCC* | R/L | 31 | 11 | -52 | 26 | 14.02 | 0.154 | 43 |  |
| iPL* | R | 39 | 35 | -58 | 38 | 10.75 | 0.122 | 19 |  |
| MTG* | R | 39 | 50 | 64 | 14 | 13.63 | 0.150 | 30 |  |
| MTG* | R | 39 | 50 | -67 | 26 | 11.24 | 0.127 | 25 |  |
| Culmen* | R/L | - | -1 | -58 | -4 | 12.91 | 0.144 | 33 |  |
| AUDIT x CUDIT x Emotion x Task Condition | | | | | | | | |  |
| iFG* | L | 9 | -40 | 5 | 29 | 7.12 | 0.085 | 35 |  |
| CUDIT x Emotion | | | | | | | | |  |
| Lentiform Nucleus/Putamen | R | - | 26 | 14 | -1 | 11.63 | 0.131 | 20 |  |
| Cerebellum | R/L | - | 2 | -73 | -22 | 10.67 | 0.122 | 20 |  |

Note: ^a^ According to the Talairach Daemon Atlas (<http://www.nitrc.org/projects/tal-daemon/>), ^b^ Based on
the Tournoux & Talairach standard brain template, * Denotes regions that overlap with and/or are proximal to
clusters found in the main analysis, BA= Brodmann’s Area

| Table S5. Brain Regions demonstrating significant Interaction Effects After Removing Smokers | | | | | | | | |  |
| --- | --- | --- | --- | --- | --- | --- | --- | --- | --- |
| Coordinates of Peak Activation^b^ | | | | | | | | |  |
| Region^a^ | Hemisphere | BA | x | y | z | *F* | Partial η^2^ | Voxels |  |
| AUDIT x Task Condition | | | | | | | | |  |
| dlPFC* | R | 8 | 29 | 35 | 44 | 19.62 | 0.224 | 114 |  |
| MFG* | R | 6 | 20 | 20 | 56 | 13.13 | 0.162 | 29 |  |
| ACC* | R/L | 24/32 | -7 | 17 | 26 | 12.03 | 0.150 | 37 |  |
| PCC/Precuneus* | R/L | 7/31 | 11 | 67 | -29 | 23.38 | 0.256 | 904 |  |
| PCC* | R | 31 | 5 | -31 | 47 | 10.80 | 0.137 | 28 |  |
| iPL* | R | 13 | 50 | -43 | 23 | 16.35 | 0.194 | 46 |  |
| iPL* | R | 40 | 35 | -49 | 41 | 13.54 | 0.166 | 26 |  |
| MTG* | R | 39 | 47 | -61 | 11 | 17.79 | 0.207 | 73 |  |
| Parahippocampal Gyrus* | L | 27 | -25 | -34 | -1 | 20.67 | 0.233 | 28 |  |
| Culmen* | R/L | - | -1 | -58 | -4 | 13.44 | 0.165 | 79 |  |
| CUDIT x Task Condition | | | | | | | | |  |
| PCC* | R | 31 | 8 | -55 | 26 | 11.67 | 0.147 | 30 |  |
| Parahippocampal Gyrus | L | 27/35 | -19 | -31 | -7 | 18.28 | 0.212 | 20 |  |
| Culmen* | R/L | - | -7 | -61 | -7 | 14.34 | 0.174 | 37 |  |
| AUDIT x CUDIT x Emotion x Task Condition | | | | | | | | |  |
| dlPFC | R | 9 | 53 | 17 | 32 | 8.27 | 0.108 | 40 |  |
| dlPFC | R | 9 | 26 | 32 | 32 | 7.63 | 0.101 | 20 |  |
| MFG | R | 6/8 | 26 | 11 | 44 | 7.32 | 0.097 | 32 |  |
| iFG/aIC | L | 13 | -40 | 20 | 8 | 9.55 | 0.123 | 46 |  |
| Precentral Gyrus | L | 4 | -55 | -10 | 32 | 8.65 | 0.113 | 48 |  |
| Precentral Gyrus | R | 6 | 53 | -7 | 29 | 8.29 | 0.109 | 39 |  |
| STG | L | 22 | -49 | -16 | -1 | 8.79 | 0.114 | 22 |  |
| Parahippocampal Gyrus | L | 36 | -34 | -25 | -13 | 12.35 | 0.154 | 26 |  |
| Cerebellum | L | - | -40 | -64 | -40 | 7.89 | 0.104 | 30 |  |

Note: ^a^ According to the Talairach Daemon Atlas (<http://www.nitrc.org/projects/tal-daemon/>), ^b^ Based on

the Tournoux & Talairach standard brain template, * Denotes regions that overlap with and/or are proximal to
clusters found in the main analysis, BA= Brodmann’s Area

| Table S6. Brain Regions demonstrating significant Interaction Effects After Removing Non-Substance Users | | | | | | | | |  |
| --- | --- | --- | --- | --- | --- | --- | --- | --- | --- |
| Coordinates of Peak Activation^b^ | | | | | | | | |  |
| Region^a^ | Hemisphere | BA | x | y | z | *F* | Partial η^2^ | Voxels |  |
| AUDIT x Task Condition | | | | | | | | |  |
| dlPFC | R | 10 | 32 | 53 | 20 | 13.85 | 0.235 | 71 |  |
| ACC* | R/L | 32 | 2 | 23 | 38 | 16.56 | 0.269 | 72 |  |
| aIC/STG | R | 22/47/13 | 53 | 11 | -1 | 13.73 | 0.234 | 38 |  |
| PCC/Precuneus/iPL/Cerebellum* | R/L | 7/31/ 39/40 | -1 | -61 | -28 | 27.15 | 0.376 | 1944 |  |
| STG* | R | 41 | 53 | -16 | 11 | 11.57 | 0.205 | 36 |  |
| Occipital Cortex | L | 19 | -34 | -79 | 29 | 12.34 | 0.215 | 32 |  |
| CUDIT x Task Condition | | | | | | | | |  |
| PCC* | R/L | 7 | 5 | -61 | 29 | 10.52 | 0.190 | 34 |  |

Note: ^a^ According to the Talairach Daemon Atlas (<http://www.nitrc.org/projects/tal-daemon/>), ^b^ Based on
the Tournoux & Talairach standard brain template, * Denotes regions that overlap with and/or are proximal to
clusters found in the main analysis, BA= Brodmann’s Area

| Table S7. Brain Regions demonstrating significant Interaction Effects with RT included in the model | | | | | | | | |  | |
| --- | --- | --- | --- | --- | --- | --- | --- | --- | --- | --- |
| Coordinates of Peak Activation^b^ | | | | | | | | |  | |
| Region^a^ | Hemisphere | BA | x | y | z | *F* | Partial η^2^ | Voxels |  |  |
| AUDIT x Task Condition | | | | | | | | |  | |
| dlPFC* | R | 8 | 29 | 35 | 44 | 17.44 | 0.185 | 93 |  |  |
| dlPFC* | L | 10 | -34 | 47 | 20 | 12.70 | 0.142 | 22 |  |  |
| MFG* | R | 6 | 20 | 20 | 56 | 12.23 | 0.137 | 25 |  |  |
| iFG* | R | 9 | 53 | 5 | 29 | 10.58 | 0.121 | 21 |  |  |
| ACC/dmPFC* | R/L | 6/32 | 2 | 11 | 44 | 13.45 | 0.149 | 28 |  |  |
| ACC/dmPFC* | R/L | 8/32 | -1 | 26 | 38 | 11.59 | 0.131 | 26 |  |  |
| Precuneus/PCC* | R/L | 7/31 | 11 | -67 | 29 | 23.08 | 0.201 | 1388 |  |  |
| iPL* | R | 40 | 35 | -49 | 41 | 14.89 | 0.162 | 31 |  |  |
| iPL* | R | 13 | 50 | -43 | 23 | 13.56 | 0.150 | 24 |  |  |
| Postcentral Gyrus* | R | 41 | 53 | -19 | 14 | 11.99 | 0.135 | 29 |  |  |
| MTG* | R | 19 | 44 | -61 | 11 | 16.69 | 0.178 | 74 |  |  |
| Supramarginal Gyrus* | R | 40 | 50 | -37 | 35 | 14.90 | 0.162 | 19 |  |  |
| Parahippocampal Gyrus* | L | 27 | -25 | -34 | -1 | 18.60 | 0.195 | 31 |  |  |
| Cerebellum* | R/L | - | -4 | -61 | -31 | 13.84 | 0.152 | 30 |  |  |
| CUDIT x Task Condition | | | | | | | | |  | |
| PCC* | R | 31 | 11 | -52 | 26 | 13.85 | 0.152 | 76 |  |  |
| Precuneus* | L | 31 | -16 | -67 | 26 | 16.34 | 0.175 | 30 |  |  |
| Precuneus* | R | 7/31 | 14 | -70 | 29 | 17.62 | 0.186 | 29 |  |  |
| iPL* | R | 39 | 35 | -58 | 38 | 12.20 | 0.137 | 28 |  |  |
| Culmen* | R/L | - | -7 | -61 | -7 | 15.36 | 0.166 | 39 |  |  |
| MTG* | R | 19 | 44 | -61 | 11 | 12.67 | 0.141 | 26 |  |  |
| MTG* | R | 39 | 50 | -67 | 26 | 11.09 | 0.126 | 23 |  |  |
| RT x Task Condition | | | | | | | | |  |  |
| Insula/iFG | R | 13 | 38 | 14 | 5 | 10.71 | 0.122 | 11^c^ |  |  |
| dmPFC | L | 6 | -7 | -10 | 53 | 13.55 | 0.150 | 11^c^ |  |  |
| AUDIT x CUDIT x Emotion x Task Condition | | | | | | | | |  |  |
| iFG* | L | 9 | -40 | 5 | 29 | 7.25 | 0.086 | 30 |  |  |

Note: ^a^ According to the Talairach Daemon Atlas (<http://www.nitrc.org/projects/tal-daemon/>), ^b^ Based on
the Tournoux & Talairach standard brain template, ^c^ Under the ClustSim generated threshold, * Denotes regions
that overlap with and/or are proximal to clusters found in the main analysis, BA= Brodmann’s Area

Supplemental Figure Legend

**Figure S1. Main effects of task condition** within clusters displayed in Figure 3. * indicates significant differences at *p*<.05.
